# *Paenibacillus* spp infection among infants with postinfectious hydrocephalus in Uganda: an observational case-control study

**DOI:** 10.1016/S2666-5247(23)00106-4

**Published:** 2023-06-19

**Authors:** Sarah U Morton, Christine Hehnly, Kathy Burgoine, Paddy Ssentongo, Jessica E Ericson, M Senthil Kumar, Cornelia Hagmann, Claudio Fronterre, Jasmine Smith, Mercedeh Movassagh, Nicholas Streck, Lisa M Bebell, Joel Bazira, Elias Kumbakumba, Francis Bajunirwe, Ronald Mulondo, Edith Mbabazi-Kabachelor, Brian K Nsubuga, Davis Natukwatsa, Esther Nalule, Joshua Magombe, Tim Erickson, Joseph Ngonzi, Moses Ochora, Peter Olupot-Olupot, Justin Onen, Peter Ssenyonga, John Mugamba, Benjamin C Warf, Abhaya V Kulkarni, Jessica Lane, Andrew J Whalen, Lijun Zhang, Kathryn Sheldon, Frederick A Meier, Julius Kiwanuka, James R Broach, Joseph N Paulson, Steven J Schiff

**Affiliations:** Division of Newborn Medicine (S U Morton MD) and Department of Neurosurgery (Prof B C Warf MD), Boston Children’s Hospital, Boston, MA, USA; Department of Pediatrics (S U Morton) and Department of Neurosurgery (Prof B C Warf), Harvard Medical School, Boston, MA, USA; Institute for Personalized Medicine, Department of Biochemistry and Molecular Biology, The Pennsylvania State University College of Medicine, Hershey, PA, USA (C Hehnly PhD, J Smith BS, K Sheldon PhD, Prof J R Broach PhD); Neonatal Unit, Department of Paediatrics and Child Health, Mbale Regional Referral Hospital, Mbale, Uganda (K Burgoine MD, P Olupot-Olupot MD); Institute of Translational Medicine, University of Liverpool, Liverpool, UK (K Burgoine); Mbale Clinical Research Institute, Mbale Regional Referral Hospital, Mbale, Uganda (K Burgoine, P Olupot-Olupot); Busitema University, Busitema, Uganda (K Burgoine, P Olupot-Olupot); Department of Medicine, The Pennsylvania State University College of Medicine, Hershey, PA, USA (P Ssentongo MD); Department of Public Health Sciences (P Ssentongo) and Department of Neurosurgery (J Lane MD), The Pennsylvania State University College of Medicine, Hershey, PA, USA; Division of Pediatric Infectious Disease, The Pennsylvania State University College of Medicine, Hershey, PA, USA (J E Ericson MD); Division of Infectious Diseases, Department of Medicine, Center for Global Health, and Medical Practice Evaluation Center and Massachusetts General Hospital and Harvard Medical School, Boston, MA, USA (L M Bebell MD); Department of Biostatistics, Harvard T H Chan School of Public Health and Department of Data Sciences, Dana Farber Cancer Institute, Boston, MA, USA (M S Kumar PhD, M Movassagh PhD); Department of Neonatology and Intensive Care, University Children’s Hospital Zurich, Zurich, Switzerland (C Hagmann MD); Department of Microbiology, Mbarara University of Science and Technology, Mbarara, Uganda (J Bazira PhD); Centre for Health Informatics, Computing, and Statistics, Lancaster University, Lancaster, UK (C Fronterre PhD); Department of Pediatrics (E Kumbakumba MBChB, M Ochora MBChB, J Kiwanuka MD†) and Department of Epidemiology (F Bajunirwe PhD) and Department of Obstetrics and Gynaecology (J Ngonzi MD), Mbarara University of Science and Technology, Mbarara, Uganda; CURE Children’s Hospital of Uganda, Mbale, Uganda (R Mulondo MD, E Mbabazi-Kabachelor MD, B K Nsubuga BSc, D Natukwatsa PGD, E Nalule CO, J Magombe PGD, T Erickson MBA, J Onen MD, J Mugamba MD, P Ssenyonga); Mulago National Referral Hospital, Makerere University, Kampala, Uganda (J Onen, P Ssenyonga MD); Division of Neurosurgery, Department of Surgery, Hospital for Sick Children, University of Toronto, ON, Canada (Prof A V Kulkarni MD); Department of Neurosurgery, Massachusetts General Hospital, Boston, MA, USA (A J Whalen PhD); Department of Pathology and Laboratory Medicine Division of Clinical Pathology, The Pennsylvania State University College of Medicine, Hershey, PA, USA (N Streck PhD); Department of Population and Quantitative Health Sciences, Case Western Reserve University, Cleveland, OH, USA (L Zhang PhD); Department of Pathology, Wayne State University School of Medicine, Detroit, MI, USA (F A Meier MD); Department of Data Sciences, N-Power Medicine, Redwood City, CA, USA (J N Paulson PhD); Department of Neurosurgery, Yale University School of Medicine, New Haven, CT, USA (Prof S J Schiff MD)

## Abstract

**Background:**

*Paenibacillus thiaminolyticus* is a cause of postinfectious hydrocephalus among Ugandan infants. To determine whether *Paenibacillus* spp is a pathogen in neonatal sepsis, meningitis, and postinfectious hydrocephalus, we aimed to complete three separate studies of Ugandan infants. The first study was on peripartum prevalence of *Paenibacillus* in mother–newborn pairs. The second study assessed *Paenibacillus* in blood and cerebrospinal fluid (CSF) from neonates with sepsis. The third study assessed *Paenibacillus* in CSF from infants with hydrocephalus.

**Methods:**

In this observational study, we recruited mother–newborn pairs with and without maternal fever (mother–newborn cohort), neonates (aged ≤28 days) with sepsis (sepsis cohort), and infants (aged ≤90 days) with hydrocephalus with and without a history of neonatal sepsis and meningitis (hydrocephalus cohort) from three hospitals in Uganda between Jan 13, 2016 and Oct 2, 2019. We collected maternal blood, vaginal swabs, and placental samples and the cord from the mother–newborn pairs, and blood and CSF from neonates and infants. Bacterial content of infant CSF was characterised by 16S rDNA sequencing. We analysed all samples using quantitative PCR (qPCR) targeting either the *Paenibacillus* genus or *Paenibacillus thiaminolyticus* spp. We collected cranial ultrasound and computed tomography images in the subset of participants represented in more than one cohort.

**Findings:**

No *Paenibacillus* spp were detected in vaginal, maternal blood, placental, or cord blood specimens from the mother–newborn cohort by qPCR. *Paenibacillus* spp was detected in 6% (37 of 631 neonates) in the sepsis cohort and, of these, 14% (5 of 37 neonates) developed postinfectious hydrocephalus. *Paenibacillus* was the most enriched bacterial genera in postinfectious hydrocephalus CSF (91 [44%] of 209 patients) from the hydrocephalus cohort, with 16S showing 94% accuracy when validated by qPCR. Imaging showed progression from *Paenibacillus* spp-related meningitis to postinfectious hydrocephalus over 1–3 months. Patients with postinfectious hydrocephalus with *Paenibacillus* spp infections were geographically clustered.

**Interpretation:**

*Paenibacillus* spp causes neonatal sepsis and meningitis in Uganda and is the dominant cause of subsequent postinfectious hydrocephalus. There was no evidence of transplacental transmission, and geographical evidence was consistent with an environmental source of neonatal infection. Further work is needed to identify routes of infection and optimise treatment of neonatal *Paenibacillus* spp infection to lessen the burden of morbidity and mortality.

## Introduction

Neonatal infections, particularly neonatal sepsis and meningitis, contribute substantially to infant mortality and morbidity worldwide.^1,2^ Neonatal sepsis is often accompanied by presumed neonatal meningitis as cerebrospinal fluid (CSF) is not always obtained to definitively assess for meningitis. A well established sequela in survivors of neonatal sepsis with presumed meningitis is postinfectious hydrocephalus (ie, infants with typical head size at birth, febrile illness or seizures during the first 28 days of life, and subsequent hydrocephalus without signs of brain malformation), which accounts for a substantial percentage of childhood hydrocephalus in low-income and middle-income countries.^3^ Hydrocephalus is the leading global indication for paediatric neurosurgery. In Uganda, postinfectious hydrocephalus was shown to account for 60% of all infant hydrocephalus cases,^4^ with one-third of patients dying and one-third of survivors exhibiting severe neurodevelopmental impairment by the age of 5 years^5^ despite successful hydrocephalus treatment. As postinfectious hydrocephalus develops at an average age of 1·3 months after neonatal infection, microbiological culture at presentation for hydrocephalus care generally fails to identify the causative pathogen in thousands of treated Ugandan infants with postinfectious hydrocephalus.^4^ Therefore, to properly prevent and treat neonatal sepsis to reduce the incidence of postinfectious hydrocephalus, a molecular diagnostic approach to rapidly detect the causative pathogen is needed.

Globally, the causal pathogens of neonatal sepsis and postinfectious hydrocephalus have not been well characterised. In neonatal sepsis, the detection of putative pathogens using microbiological culture of blood and CSF samples (the gold standard for diagnosing neonatal sepsis and meningitis) can be as low as 0·8%.^6,7^ Only 12·8–32·5% of infants had a positive blood culture in two case series^8,9^ with neonatal sepsis in Uganda. The most common pathogens isolated were *Staphylococcus aureus* and *Escherichia coli*.^8,9^ In a previous analysis of 100 Ugandan infants with postinfectious hydrocephalus following presumed neonatal sepsis, we used targeted 16S rRNA gene amplicon sequencing to identify *Paenibacillus* spp as the dominant bacteria associated with postinfectious hydrocephalus.^8^ Furthermore, three isolates of *Paenibacillus thiaminolyticus* were recovered from the CSF of patients with postinfectious hydrocephalus, which showed substantial lethality in mice.^10,11^

*Paenibacillus* spp had been isolated in case reports of neonatal sepsis, but not as a predominant pathogen.^12^ This study was undertaken with the aim to address several key issues about *P thiaminolyticus* spp infection in infants from Uganda: (1) is there evidence of in utero or peripartum transmission?; (2) what is the prevalence in neonates with sepsis?; (3) how many neonates with sepsis develop postinfectious hydrocephalus?; (4) which bacteria are the major causes of postinfectious hydrocephalus?; (5) what is the prevalence of *Paenibacillus* spp in a larger group of infants with postinfectious hydrocephalus?; and (6) is there geographical clustering of infections? To address these questions, we studied three Ugandan cohorts of mother–newborn pairs, neonates with sepsis, and infants with hydrocephalus.

## Methods

### Study design and participants

In this observational case-control study, we recruited participants belonging to three separate cohorts: mother–newborn pairs, with and without maternal fever (mother–newborn cohort); neonates (aged ≤28 days) with sepsis (neonatal sepsis cohort); and infants (aged ≤90 days) with hydrocephalus, with and without a history of neonatal sepsis or meningitis (hydrocephalus cohort).

For the mother–newborn pair study, we recruited labouring expectant mothers from the Mbarara Regional Referral Hospital (Mbarara, Uganda) and Mbale Regional Referral Hospital (Mbale, Uganda) between May 20, 2016 and May 8, 2017, as previously described.^13,14^ We recruited women (aged ≥18 years) who were in labour and able to provide informed written consent in a local language. We included women who delivered at term (>37 weeks estimated gestational age). Women were excluded if there was known intrauterine fetal death, pre-eclampsia, emergency delivery impeding sample collection, antepartum hemorrhage, no telephone number available for post-discharge contact, or domicile (>10 km) from the hospital.

For the neonatal sepsis study, we recruited neonates from Mbarara Regional Referral Hospital and Mbale Regional Referral Hospital between Jan 13, 2016 and Oct 2, 2019. We included neonates who presented with clinical sepsis. Sepsis was defined as the presence of one of the following three combinations of signs: (1) axillary temperature >37·5°C, lethargy, and poor feeding, (2) axillary temperature <35·5°C, lethargy, and poor feeding, or (3) full fontanelle or seizures, axillary temperature >37·5°C, and poor feeding. Neonates were excluded from recruitment if they had major congenital abnormalities, had a history of perinatal asphyxia, or had received antibiotics for more than 24 h before recruitment. Six neonates from the mother–newborn pair study were also recruited to the neonatal sepsis study.

For the hydrocephalus study, we recruited infants from the CURE Children’s Hospital of Uganda (Mbale, Uganda) between Feb 22, 2016 and April 30, 2019. We included infants who were younger than 90 days, and had postinfectious hydrocephalus (ie, typical head size at birth, febrile illness or seizures during the first 28 days of life, and subsequent hydrocephalus without stigmata of a brain malformation such as Dandy–Walker malformation on cranial imaging) or non-postinfectious hydrocephalus (ie, born with hydrocephalus or with stigmata of a brain malformation on cranial imaging). We excluded infants who had a previous surgery on the nervous system (eg, ventriculoperitonieal shunt, third ventriculostomy, or myelomeningocele closure), or evidence of communication of nervous system with skin (eg, meningocele, encephalocele, dermal sinus tract, or fistula). Cases (ie, infants with postinfectious hydrocephalus) and controls (ie, infants with non-postinfectious hydrocephalus) were not matched on any demographic or clinical criteria other than the inclusion criteria. Full inclusion and exclusion criteria and informed consent requirements for all studies are in the appendix (pp 1–2).

Ethical approval was granted by the CURE Children’s Hospital of Uganda Institutional Review Board (CCHUREC/02/018), Mbarara University of Science and Technology Research Ethics Committee (reference number 12/11–15), Ugandan National Council on Science and Technology (reference number HS/1963), Pennsylvania State University (reference numbers STUDY0004199 and 10418), and the Yale University Institutional Review Boards (2000033405).

### Procedures

For the mother–newborn pair study, we collected four sample types: intrapartum maternal blood (1 mL), intrapartum vaginal swab, postpartum cord blood (1 mL), and fetal-side placenta.

For the neonatal sepsis study, we collected blood and CSF samples. We performed blood culture using the BD Bactec system (Beckton Dickenson and Company, Franklin Lakes, NJ, USA) with at least 1 mL of blood, as per the manufacturer’s instructions. Blood cultures were ideally collected before antibiotic therapy was initiated at a referring centre but most neonates received antibiotics at other health facilities before admission to the referral hospitals.

For the hydrocephalus study, we collected CSF samples at the time of surgery during either shunt placement or endoscope insertion (appendix p 18) as previously described,^15^ and participants did not have indwelling CNS hardware before sampling. Description of CT scan scoring is available in the appendix (pp 5–6).

Samples were divided for fresh freezing or placement into DNA/RNA Shield (Zymo Corporation, Tustin, California, USA), and all specimens were then stored at −80°C.

### DNA sequencing and 16S rDNA amplicon analysis

Samples from all cohorts were analysed using targeted quantitative PCR (qPCR) to detect *Paenibacillus* spp. Samples from the hydrocephalus cohort were also analysed using unbiased 16S rDNA metagenome sequencing.

The sequencing methods are described in the appendix (pp 2–4). Nucleic acid extraction, 16S amplification, and library preparation were performed as previously described.^15^ 16S rRNA DNA sequences were processed using Qiime2 (version 2021.4).^16^ Amplicon sequence variant count matrices were generated and normalised using the metagenomeSeq R Bioconductor package (version 1.32.0).^17,18^

### Outcomes

The primary outcome was genus-level 16S rRNA gene counts, assessed as absolute and relative abundance, both per participant and in participant groups. Secondary outcomes were species-level 16S rRNA gene counts, and qPCR counts for *Paenibacillus* spp.

### Statistical analysis

Two-tailed Student’s *t* test to compare means or Wilcoxon rank-sum tests to compare medians of two groups were used for continuous variables. Statistical significance level was set at p<0·05. Patients with missing data were included and were noted in relevant tables. All analyses were performed with the R statistical language (version 3.0.6). Comparison of the geographical clustering of postinfectious hydrocephalus and non-postinfectious hydrocephalus and *Paenibacillus* positive and negative participants were performed using Ripley’s K function.^19^ For privacy reasons, village centroids were mapped within an 11 km square area, and participants of villages with less than 500 people were omitted.

### Role of the funding source

The funders of the study had no role in study design, data collection, data analysis, data interpretation, or writing of the report.

## Results

**Figure 1:**
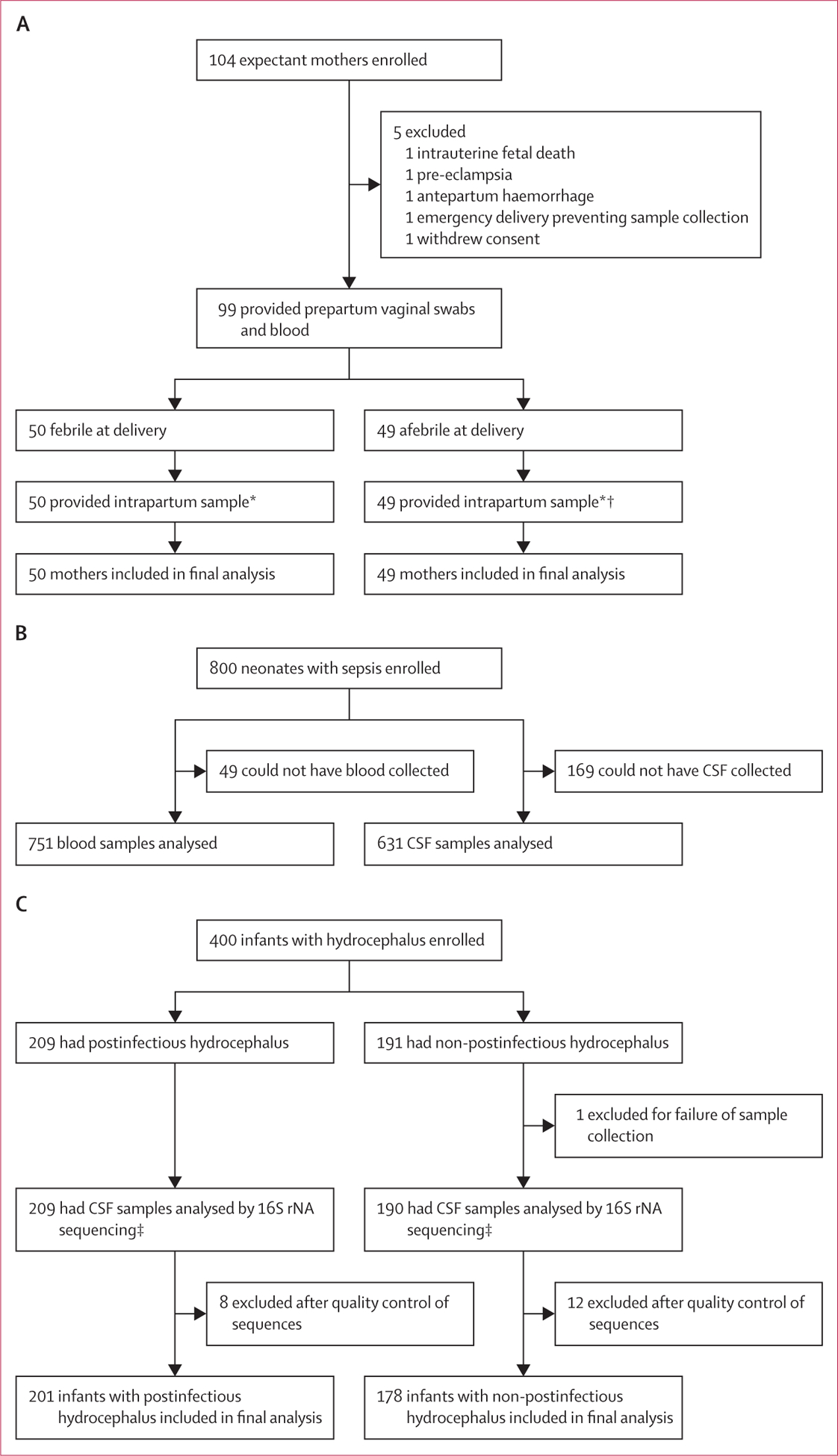
Participant flow diagrams (A) Mother–newborn cohort. (B) Neonatal sepsis cohort. (C) Hydrocephalus cohort. CSF=cerebrospinal fluid. *Peripartum vaginal swab, maternal blood, and placenta sample. †Three placentas not available. ‡All 209 postinfectious hydrocephalus and 191 non-postinfectious hydrocephalus cases were included in quantitative PCR analysis and final designation of *Paenibacillus* spp.

Details of participant enrolment and inclusion are shown in figure 1. We enrolled 400 infants with hydrocephalus, including 209 (52%) patients with postinfectious hydrocephalus and 191 (48%) patients with non-postinfectious hydrocephalus (appendix p 18). Within the postinfectious hydrocephalus group, the mean age of infants presenting with signs of infection by history was 8·0 days (SD 7·0), and the mean interval between first infection and CSF sampling with hydrocephalus was 51·5 days (18·5). Patients with postinfectious hydrocephalus were on average older (61·8 days [SD 17·4]; p<0·0001) and had higher white blood cell counts in both blood (10·9 cells × 103 per μL [3·2]; p<0·0001) and CSF (42·5 cells × 103 per μL [74·0]; p<0·0001) samples than the non-postinfectious hydrocephalus group. Patients with postinfectious hydrocephalus were more likely to have cytomegalovirus detected in a CSF sample (12·4% [26 of 209 patients]) than the non-postinfectious hydrocephalus group (0·5% [1 of 190 patients]; p<0·0001). Patients with postinfectious hydrocephalus also had lower haemoglobin concentration (10·6 g/dL [1·6]) than the non-postinfectious hydrocephalus group (13·6 g/dL [2·9]; p<0·0001].^13^ The two groups were not different with regard to sex, HIV exposure, and detection of cytomegalovirus in the blood (appendix pp 7–8). CT scoring was based on the presence of loculation, calcification, debris within fluid collections, or abscess formation, and postinfectious hydrocephalus was associated with more severe brain CT scores, with 122 (62%) of 196 patients having scores of 2 or more compared with ten (6%) of 178 patients in the non-postinfectious hydrocephalus group (odds ratio 29·2, 95% CI 14·6–58·5; p<0·0001). These cohort differences were consistent among the samples that remained after quality control filtering (appendix p 9). Of the samples that were *Paenibacillus* spp positive on qPCR, the infants with postinfectious hydrocephalus were around a week younger at sample collection (57·5 days [SD 16·6] *vs* 65·5 days [17·1]) and had a higher white blood cell count in the blood (11·6 cells × 103 [3·6] *vs* 10·4 cells × 103 [2·8]) and CSF (66·7 [85·5] *vs* 20·5 [53·9] cells per μL)compared with non-postinfectious hydrocephalus patients (appendix p 10).

Per sample, the number of features generally increased with the number of reads in both groups, regardless of sequencing batch (appendix p 18). There were no systematic differences in the intra-sample diversity of bacterial genera by group as measured by the Shannon index, and inter-sample diversity was largely overlapping for the cohorts (appendix p 19). The patterns of inter-sample diversity remained true after leveraging novel approaches to account for spurious technical artifacts and when samples with *Paenibacillus* spp detected by qPCR were removed (appendix pp 4–5).

**Figure 2:**
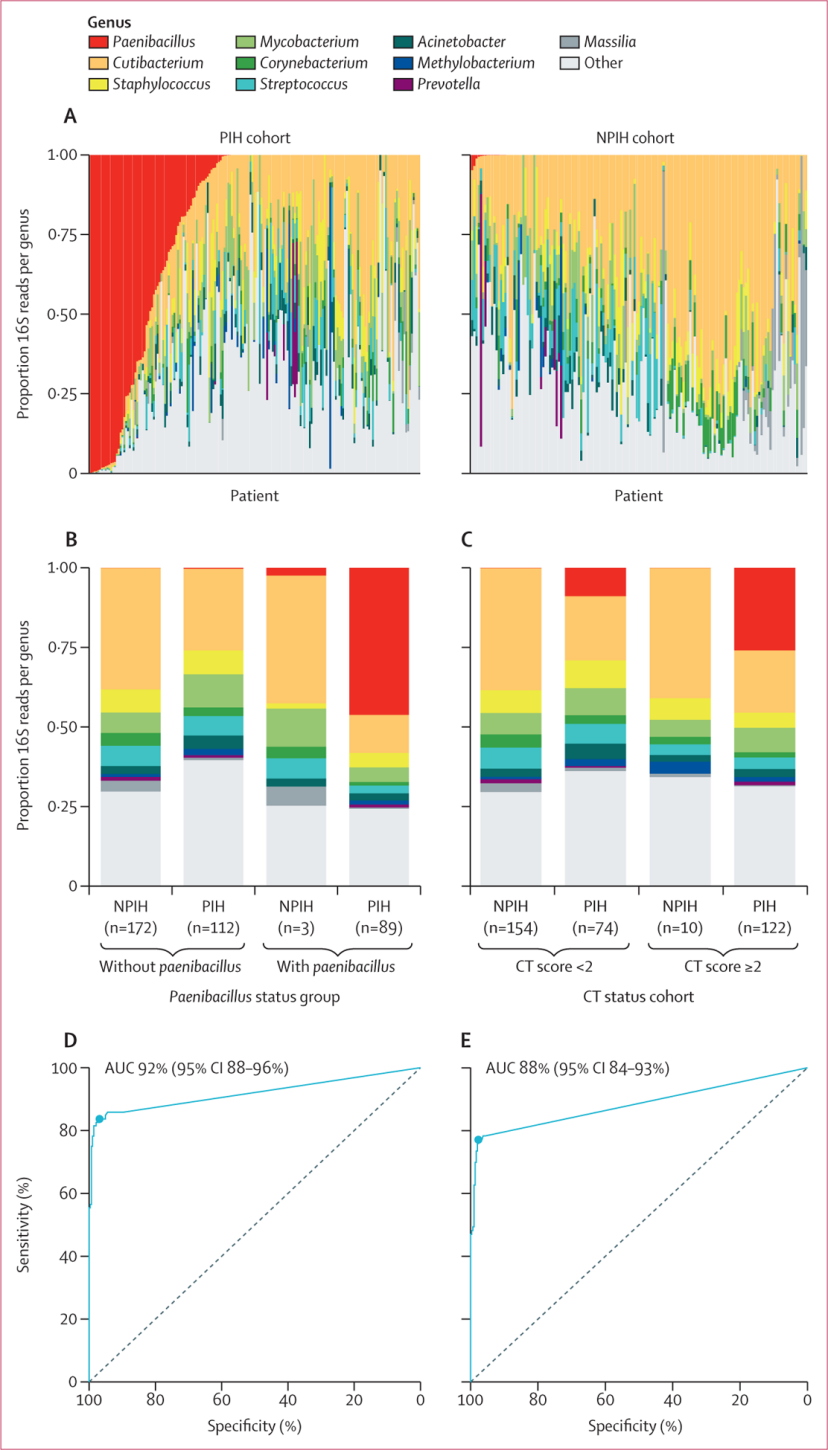
Paenibacillus spp associated with PIH using 16S metagenome sequencing (A) Proportions of 16S reads mapped to the ten most common genera by patient for the PIH (left) and NPIH (right) groups. (B, C) Proportion of 16S reads mapped to the ten most common genera by PIH (B) or NPIH (C) status. (D, E) Receiver operating characteristic curves were used to optimise the sensitivity and specificity of 16S *Paenibacillus* diagnosis with the gold standard quantitative PCR of 54·5 *Paenibacillus* spp and 32·5 *Paenibacillus thiaminolyticus* reads. AUC=area under the curve. PIH=postinfectious hydrocephalus. NPIH=non-postinfectious hydrocephalus.

The proportion of reads per bacterial genus were calculated for individual infants with hydrocephalus and for the two groups (figure 2; appendix p 11). For postinfectious hydrocephalus patients, *Paenibacillus* and *Cutibacterium* were the most common bacterial genera, followed by *Mycobacterium*, *Staphylococcus*, and *Streptococcus*. Within the non-postinfectious hydrocephalus group, the overall proportion was similar to the postinfectious hydrocephalus cohort aside from *Paenibacillus*: *Cutibacterium* was the most common bacterial genera, followed by *Staphylococcus*, *Streptococcus*, and *Mycobacterium*. Differential 16S abundance of bacterial genera identified *Paenibacillus* and *Rickettsialis* spp as the most highly enriched bacterial genera in CSF from patients with postinfectious hydrocephalus, whereas *Cutibacteria* and *Corynebacteria* were the most highly enriched in CSF from patients with non-postinfectious hydrocephalus (appendix p 11). Both counts and proportion of *Paenibacillus* spp were higher in the postinfectious hydrocephalus group than in the non-postinfectious hydrocephalus group (appendix p 20). *Paenibacillus* spp counts did not correlate with age at initial infection (ρ=–0·01, p=0·85) or age at sampling (ρ=–0·08, p=0·11), but were negatively correlated with the number of days between initial infection and sample collection (ρ=–0·36, p<0·0001; appendix p 20). The only species enriched in postinfectious hydrocephalus were *Paenibacillus popilliae* and *P thiaminolyticus* spp (appendix p 12). Despite increased abundance at the genus level, *Rickettsialis* spp were not differentially abundant, indicating that those reads are not associated with infection. The top differentially abundant species in non-postinfectious hydrocephalus CSF were *Corynebacterium kroppenstedtii, Cutibacterium granulosum, Sphingomonas leidyi,* and *Hydrogenophilus islandicus*.

**Figure 3:**
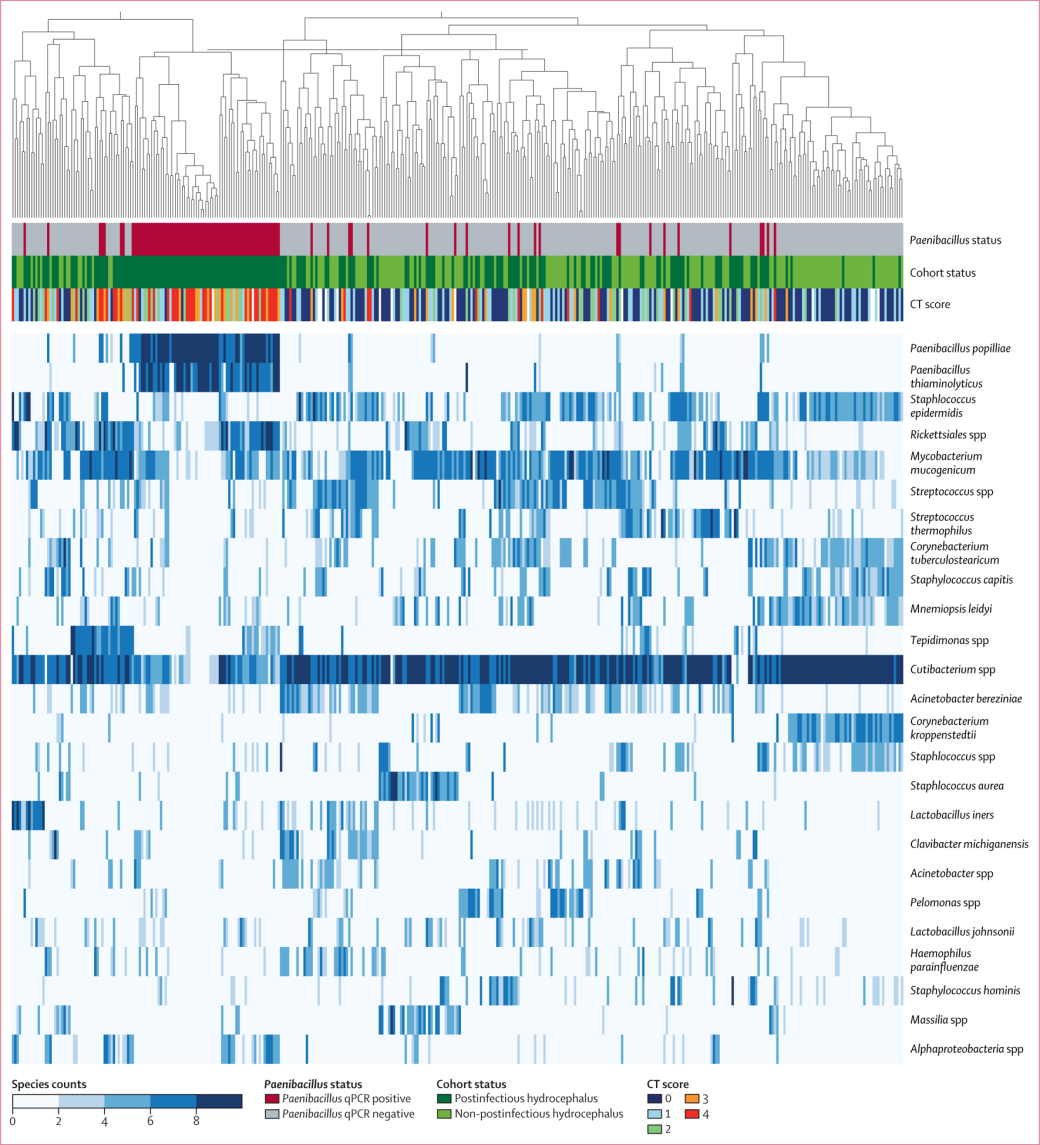
Paenibacillus spp positive cases cluster by postinfectious hydrocephalus group and CT scores clustered by bacterial species reads Unsupervised hierarchical clustering of samples by 16S reads mapped at the species level. Columns are coloured by hydrocephalus group and CT scores but group and CT score data were not used in clustering. *Paenibacillus* spp quantitative PCR status is positive or negative based on genus-level qPCR detection of *Paenibacillus*. Group status refers to postinfectious hydrocephalus or non-postinfectious hydrocephalus grouping. CT scores reflect the quantitative ranking of abnormalities on brain CT, with 0 being minimally affected and 4 being the most severely affected.

Hierarchical clustering of CSF samples by 16S bacterial species revealed two clusters that primarily contained postinfectious hydrocephalus CSF samples and were high in *Paenibacillus* spp and low in counts for most other genera or species (figure 3). The postinfectious hydrocephalus cluster had moderate counts mapped to *Mycobacterium mucogenicum*, although counts for this species were distributed through a broad range of sample clusters*. Rickettsiales* spp counts were higher than in the postinfectious hydrocephalus clusters. CSF samples did not cluster based on the presence or absence of cytomegalovirus in blood or CSF (data not shown). Patients with high *Paenibacillus* counts were generally also those with the most severe brain CT findings scores of 2 and above (figure 3).

We performed qPCR for *Paenibacillus spp* and *P thiaminolyticus* to independently assess their prevalence. A 24% (94 of 399 patients; 95% CI 19·48–28·03%) *Paenibacillus spp* prevalence was detected overall with 44% (91 of 209 patients; 37–51%) of postinfectious hydrocephalus cases and 2% (3 of 194 patients; 3–4%) of non-postinfectious hydrocephalus cases. Using the species-specific probes, a 21% (85 of 399 patients; 17–26%) *P thiaminolyticus* prevalence was detected overall; all were postinfectious hydrocephalus cases (85 patients [41%] of 209; 95% CI 35–49%). There was a 99% (84 of 85 patients) concordance of those detected with both the species-specific probes compared with genus; *P thiaminolyticus* quantities decreased with age (p=0·029), but the *Paenibacillus* spp quantities did not decrease with age (appendix p 21). In our previous analysis,^15^ we optimised 16S count cutoffs with receiver operating characteristic curves based on postinfectious hydrocephalus and non-postinfectious hydrocephalus status, but here we used the qPCR detection to optimise the 16S count cutoff to 54·5 (area under the curve 92%; 95% CI 88–96%) for genus detection and 32·5 (88%; 84–93%) for species-specific detection (figure 2; appendix p 20). Accuracy of qPCR and 16S detection was 0·94 (Cohen’s κ; 95% CI 0·91–0·96) for *Paenibacillus* spp and 0·93 (0·90–0·95) for *P thiaminolyticus* (appendix p 13).

**Figure 4:**
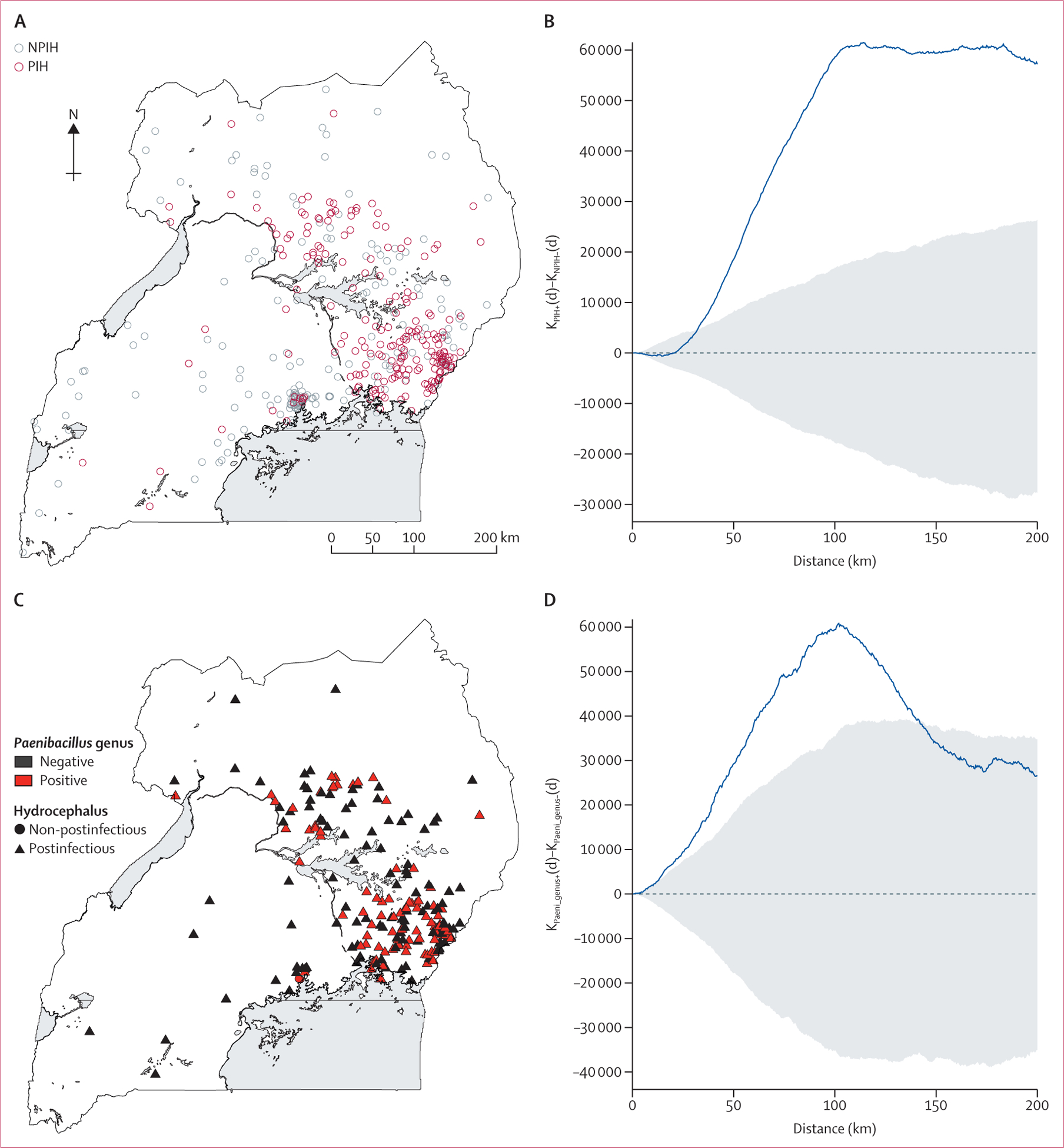
Spatial distribution of infants with hydrocephalus by cause and *Paenibacillus* status (A) Distribution of non-postinfectious hydrocephalus (black circles) and postinfectious hydrocephalus (red circles) cases in Ugandan villages, mapped within 11 km squares. Postinfectious hydrocephalus cases cluster in eastern and north-central regions whereas non-postinfectious hydrocephalus cases are more dispersed. (B) Ripley’s K function for the difference in postinfectious hydrocephalus and non-postinfectious hydrocephalus case locations (black line) shows spatial aggregation of postinfectious hydrocephalus above a 35 km scale, defined by the 95% confidence bounds from Monte Carlo simulations (grey). (C) Distribution of *Paenibacillus* spp qPCR positive non-postinfectious (red circles) and postinfectious cases (red triangles) and *Paenibacillus* spp qPCR negative non-postinfectious (black circles) and postinfectious (black triangles) cases. (D) Ripley’s K function for the difference in *Paenibacillus* spp positive and negative case locations (black line) shows spatial aggregation of *Paenibacillus* spp cases at a 5–150 km scale, defined by the 95% confidence bounds from Monte Carlo simulations (grey). PIH=Postinfectious hydrocephalus. NPIH=Non-postinfectious hydrocephalus.

We examined the spatial distribution of cases based upon the geographical location of patient villages stratified by either cause, postinfectious hydrocephalus or non-postinfectious hydrocephalus, or all hydrocephalic infants based on *Paenibacillus* status, to represent the population at risk of developing either postinfectious hydrocephalus or *Paenibacillus* spp infection (figure 4). Postinfectious hydrocephalus cases clustered above the degree of spatial aggregation from the non-postinfectious hydrocephalus cases above 35 km. Similarly, postinfectious hydrocephalus cases that were *Paenibacillus* spp positive clustered above the degree of spatial aggregation from the postinfectious hydrocephalus *Paenibacillus* negative cases between approximately 5 km and 150 km. Ripley’s K function analysis of nearest neighbours confirmed non-random clustering of postinfectious hydrocephalus and postinfectious hydrocephalus *Paenibacillus* positive cases compared with the 95% confidence estimate of random clustering from Monte Carlo permutation of case-control status in 1000 trials.

To investigate the potential vertical maternal transmission of *Paenibacillus* spp or *P thiaminolyticus*, we evaluated prepartum vaginal swabs and maternal blood, and postpartum placental specimens and cord blood, collected in a population of 99 mother–newborn pairs from Uganda. We did not detect any evidence of *Paenibacillus* spp or *P thiaminolyticus* by qPCR in any of the samples (table). The prevalence of *P thiaminolyticus* in neonatal sepsis was determined for 800 infants with neonatal sepsis, of whom 94% (751 of 800 patients) had blood collected and 79% (631 of 800 patients) had CSF collected. Overall, 6% (37 of 631 neonates) with sepsis had *Paenibacillus spp* or *P thiaminolyticus* detected by qPCR in either CSF or blood (table). There was a 92% (22 of 24 patients) concordance between species and genus-level detection (appendix p 14).

11 (1%) of 800 newborns with neonatal sepsis developed postinfectious hydrocephalus, and, of those, five (56%) of nine neonates had evidence of a *P thiaminolyticus* infection, whereas two neonates did not have CSF available (appendix pp 13–15). Of the neonates with evidence of CNS *P thiaminolyticus* infection, five (14%) of 37 neonates developed postinfectious hydrocephalus and three neonates were recruited into the hydrocephalus study as linkage cases under 90 days of age requiring surgical treatment for hydrocephalus. Two of the three linkage cases between the studies had *P thiaminolyticus* still detected in their CSF at the time of surgery for postinfectious hydrocephalus. Among the five others, four of the neonatal sepsis patients were not included in the hydrocephalus study because they did not meet the inclusion criteria and one neonate did not return for surgery after referral. Not one of the patients (0 of nine patients) with positive genus-level qPCR detection of *Paenibacillus* without species-level detection of *P thiaminolyticus* developed postinfectious hydrocephalus.

**Figure 5:**
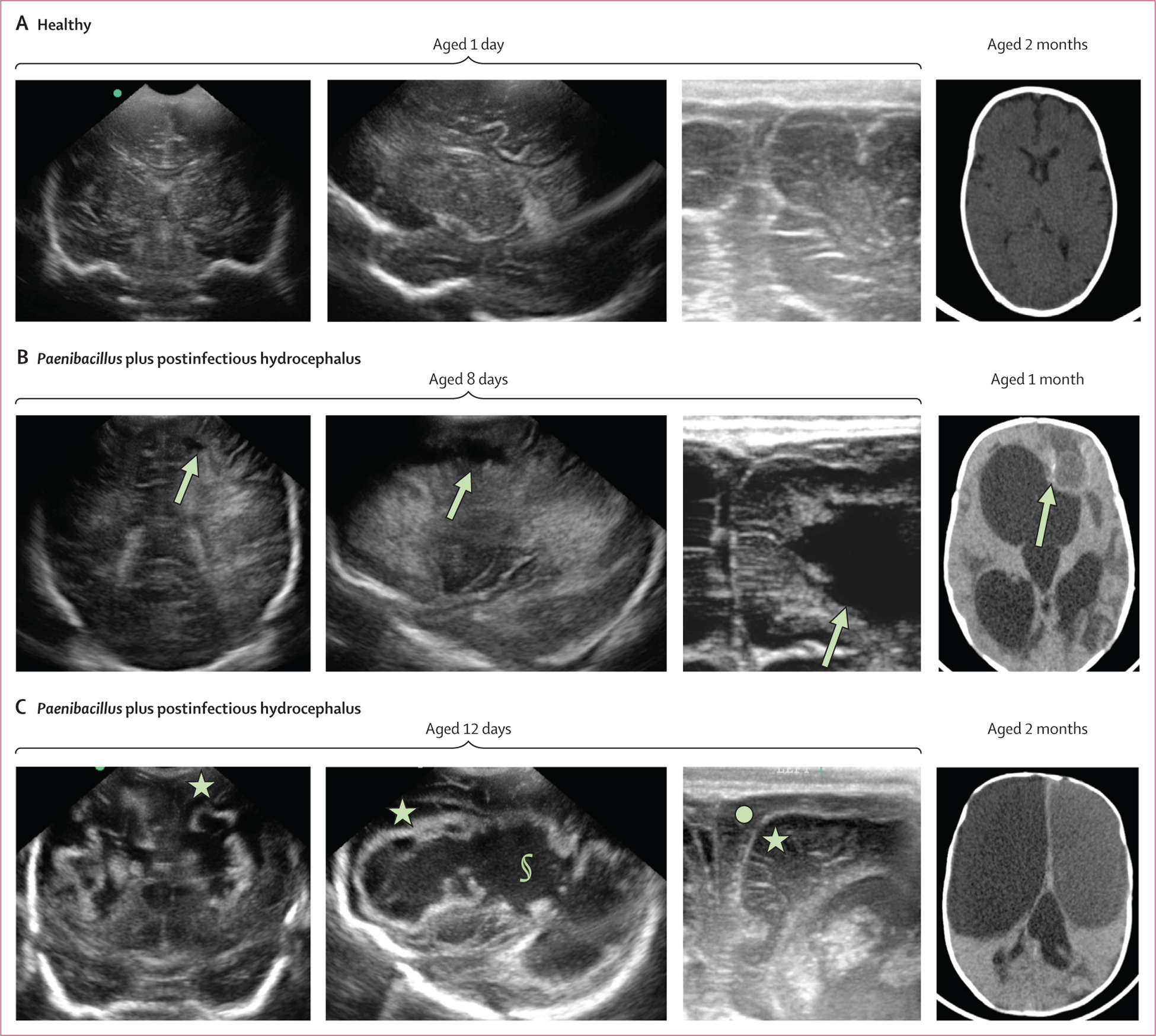
Imaging of neonatal *Paenibacillus thiaminolyticus* sepsis cases that subsequently developed postinfectious hydrocephalus 1 month=28 days. Representative cranial ultrasound from coronal, parasagittal and coronal views, and axial CT images, shown from left to right. A) Cranial ultrasound of a healthy neonate and CT image of a healthy 2-month-old infant are shown (reproduced from https://radiopaedia.org).^20^ B) 8-day old neonate (patient 1) with *P thiaminolyticus* CNS infection. Cranial ultrasound shows abnormal white matter and cortex echogenicity, extensive cortical and white matter injury from frontal to occipital lobes bilaterally, periventricular white matter injury, and a left frontal cortex and subcortical cystic lesion (green arrow). CT image of the same patient at 1 month before surgery for postinfectious hydrocephalus shows enlargement of the cyst with abscess formation (green arrow). C) 12-day-old neonate (patient 2) with *P thiaminolyticus* CNS infection. Cranial ultrasound shows cortical and subcortical white matter lesions with cystic evolution (white star), ventricular fluid with loculation, thickened hyperechogenic ventricular ependymal lining (S symbol), hyperechogenic gyri and sulci, and hyperechogenic extracerebral space (green circle). CT image of the same patient at 2 months of age before surgery for postinfectious hydrocephalus shows destruction of the bilateral frontal lobes, and residual ventricular debris.

A male infant (patient 1) was admitted to Mbale Regional Referral Hospital 8 days after home vaginal birth at 43 weeks’ gestation to a 29-year-old gravida three woman who was HIV negative and reported no intrapartum fevers but was febrile during labour. No resuscitation at birth was required and the patient had been exclusively breastfeeding at home. 6 days after birth, he presented to a lower-level care facility with failure to breastfeed, neck stiffness, and a temperature of 36·7°C prompting intravenous ampicillin and gentamicin treatment. After 2 days, he was referred to Mbale Regional Referral Hospital, where he presented with a 38·3°C fever, irritability, seizures, and a bulging fontanelle (appendix p 16). He was given oxygen via nasal cannula, dextrose 10% 2·5 mL/kg, phenobarbitone 20 mg/kg (for 5 days), ceftriaxone 100 mg/kg, and intravenous fluids. A cranial ultrasound showed cerebritis and a left frontal lobe cystic lesion (figure 5). Blood culture, HIV, and malaria testing at recruitment were negative. After 7 days of therapy with ceftriaxone 80 mg/kg once a day and gentamicin 5 mg/kg once a day, the infant was still irritable and febrile. He was then given ceftriaxone 80 mg/kg and amikacin 15 mg/kg for 14 days, during which the fever resolved, and he improved clinically. Due to the suspected brain abscess, he was discharged with a 14-day course of oral metronidazole, amoxicillin, and ciprofloxacin. He was referred to the CURE Children’s Hospital for enlarging head size and, at the age of 44 days, a follow-up CT scan showed hydrocephalus, left frontal lobe abscess with calcification, and multiple areas of white and grey matter injury (figure 5). Research samples were qPCR positive for *Paenibacillus* spp at ages 8 days and 44 days.

Another male infant (patient 2) was admitted to Mbale Regional Referral Hospital 12 days after a vaginal birth to a 23-year-old gravida two woman at a health centre by vaginal delivery. His mother was HIV negative, reported five intrapartum episodes of fever, and was febrile during labour. He required no resuscitation at birth and had been exclusively breastfeeding at home. 11 days after birth, he presented to a lower-level care facility with difficulty breathing and poor feeding prompting intravenous ampicillin and gentamicin treatment for 1 day before referral to Mbale Regional Referral Hospital. At Mbale Regional Referral Hospital admission, he presented with a 37·0°C temperature, irritability, active seizures, severe jaundice, and a bulging fontanelle (appendix p 16). He was given oxygen therapy, intravenous fluids, phototherapy, and continued intravenous ampicillin 50 mg/kg and gentamicin 3 mg/kg. Blood culture, HIV, and malaria testing at admission were negative. 12 h after admission, his seizures worsened, and he had abnormally extended posture. He was given phenobarbitone 30 mg/kg for seizures and 50 mg/kg cefotaxime twice a day and gentamicin 3 mg/kg once a day. The cranial ultrasound showed cortical and subcortical white matter lesions with cystic changes, enlarged lateral ventricles with debris and loculated fluid, and thickened hyperechogenic ventricular ependymal lining (figure 5). After 4 days of therapy with cefotaxime 50 mg/kg twice a day and gentamicin 3 mg/kg once a day, the infant remained comatose, hypotonic, and febrile. He was then given ceftriaxone 50 mg/kg and amikacin 10 mg/kg for 14 days and began to breastfeed but remained hypotonic. During a follow-up visit at 9 weeks, the infant was still having one to two seizures a day and had an increased head circumference prompting a referral to the CURE Children’s Hospital of Uganda. At 73 days, a CT scan revealed hydrocephalus and bilateral frontal lobe destruction (figure 5). Research samples were qPCR positive for *Paenibacillus* at ages 12 days and 3 months (71 days).

## Discussion

In this study, we provide evidence that *Paenibacillus* spp was the dominant cause of postinfectious hydrocephalus in 209 Ugandan infants, with a 44% (95% CI 37–51) prevalence. *Paenibacillus* spp was not detected in a study of 99 mother–newborn pairs but, instead, it was detected in 6% of a cohort of neonates with clinical sepsis. Of the neonatal sepsis group, a substantial cause of subsequent postinfectious hydrocephalus in survivors with CSF available was *P thiaminolyticus* (5 [56%] of 9 neonates) and we were able to follow up several patients with proven *P thiaminolyticus* sepsis and meningitis through to subsequent hydrocephalus; indeed, they showed active brain infection with *P thiaminolyticus* 1–3 months after initial neonatal sepsis. No detection in any mother–newborn pairs and the age of the neonates with *Paenibacillus* spp infection is consistent with a postnatally acquired infection. *P thiaminolyticus* infections were geographically clustered, further suggesting the likely role of environmental risks. Further investigation including environmental sampling is needed to assess potential sources.

Reports of *Paenibacillus* infection have been exceedingly rare,^21,22^ but two cases of *P thiaminolyticus* brain infections in neonates have been reported in the USA.^23,24^ We previously reported *Paenibacillus* in a substantial proportion of infants with postinfectious hydrocephalus with a presumed history of meningitis in Uganda.^8^ Our findings of 37 (6%) of 631 cases of *Paenibacillus* neonatal sepsis is the largest known cluster of cases so far described.^25^ *P thiaminolyticus* is a facultative anaerobe that can be difficult to recover in culture^8^ and, at present, no molecular diagnostics are regularly available at the Ugandan study sites. Blood (four [1%] of 751 patients) was less likely than CSF (35 [6%] of 631 patients) to yield positive results for *Paenibacillus* spp infection, suggesting that it would be best to both optimise culture in blood and implement molecular diagnostics in parallel to improve identification.

Organisms commonly associated with postinfectious hydrocephalus elsewhere are known to establish prolonged infection,^26^ and our findings are similar. Of the 11 patients with neonatal sepsis who developed postinfectious hydrocephalus, many (five [45%] of 11 patients) had *P thiaminolyticus* infections, including the two infants recruited into both cohorts and in both the neonatal sepsis and hydrocephalus cohorts (patients 1 and 2). Both infants had brain destruction and abscess formation on brain imaging. We previously detected an active immune response in the first 26 hydrocephalic infants with *P thiaminolyticus*, further supporting the presence of an active infection for up to 3 months.^27^ Inadequate treatment of bacterial infections is known to increase the risk of neurological sequelae,^28^ but these patients were treated at expert neonatal units in Mbale and Mbarara. The finding of residual brain infection and evolving hydrocephalus in infants following prolonged courses of intravenous antibiotic therapy for neonatal sepsis indicates that more effective protocols for identifying, preventing, and treating this organism are required and identifying the source of infection can aid in possible interventions to prevent infection. Optimal antibiotic therapy is unknown but resistance to ampicillin and vancomycin is common.^10^ Ceftriaxone or meropenem could be effective more often but resistance to these antibiotics is possible.^12^

Limitations to this study include the use of 16S rRNA gene sequencing, which could fail to detect non-bacterial pathogens. The small size of the mother–newborn cohort limits the ability to identify vertical transmission or vaginal colonisation. The distinction between non-postinfectious hydrocephalus versus postinfectious hydrocephalus was made based on clinical history; some infants might have been misclassified.

In conclusion, *Paenibacillus* spp are an important but previously unappreciated cause of neonatal sepsis and postinfectious hydrocephalus in Uganda. Extensive clinical culture at point of care have uniformly failed to isolate *Paenibacillus* spp in neonatal sepsis or postinfectious hydrocephalus patients in Uganda, reflective of how difficult this organism is to study using standard clinical microbiological approaches.^10,15^ We isolated three strains and are now working towards isolating multiple strains of this *P thiaminolyticus* organism from infected infants to define the spectrum of antibiotic sensitivity and resistance patterns. Nevertheless, our experience in these 1400 patients suggests that *P thiaminolyticus* sepsis in neonates can lead to a fulminant and persistent brain infection, despite completion of standard antibiotic therapy for neonatal sepsis, highlighting the need to develop effective treatment guidelines. Identifying the source and routes of these infections is crucial for preventing neonatal sepsis and the consequences of postinfectious hydrocephalus due to *P thiaminolyticus*.

## Data sharing

All relevant code is available online (https://github.com/Schiff-Lab).

## Supplementary Material

1

## Figures and Tables

**Table:** Prevalence of *Paenibacillus* genus and *Paenibacillus thiaminolyticus*

	Mother-newborn (n=800)	Neonatal sepsis (n=631 CSF samples; n=751 blood samples)*		Infants with postinfectious hydrocephalus (n=209)†	
	Genus or species	Genus	Species	Genus	Species
Maternal blood	0	NA	NA	NA	NA
Vaginal swab	0	NA	NA	NA	NA
Placental specimen	0	NA	NA	NA	NA
Cord or neonatal blood	0	1 (<1%)	3 (<1%)	NA	NA
Cerebrospinal fluid	NA	33 (5%)	24 (4%)	91 (44%)	85 (41%)

Data are n (%). Paenibacillus genus and Paenibacillus thiaminolyticus were not detected in maternal blood, vaginal swabs, or placental specimes from mother-newborn pairs. CSF= cerebrospinal fluid. NA=not applicable.

* Total of 37 neonates had Paenibacillus detected: 5 only by species quantitative PCR (qPCR), 11 only by genus qPCR.

† Total of 94 infants had Paenibacillus detected: 3 only by species qPCR, 9 only by genus qPCR.

## Research in context

### Evidence before this study

Hydrocephalus is the most common indication for neurosurgery in children worldwide, with approximately half of cases occurring after a previous clinical infection although a specific pathogen is often not known. We searched PubMed on Dec 12, 2021, using the search terms “neonatal sepsis” and “Uganda”. We searched for retrospective studies published between Jan 1, 2010, and Dec 1, 2021, with no language restrictions, and found 11 relevant publications. Previous studies found a specific pathogen that has been identified in less than half of patients with neonatal sepsis in Uganda, the most common antecedent of postinfectious hydrocephalus, limiting effective prevention and treatment. *Paenibacillus thiaminolyticus* was identified as an important cause of postinfectious hydrocephalus among Ugandan infants in 2020, presumably as a consequence of previous neonatal sepsis and meningitis. Patients with postinfectious hydrocephalus with *Paenibacillus* spp show severe inflammation in their cerebrospinal fluid and are at high risk for needing surgical intervention. However, the prevalence of *Paenibacillus* spp among febrile women and neonates with sepsis has not been determined.

### Added value of this study

We confirm *Paenibacillus* spp as a pathogen responsible for neonatal postinfectious hydrocephalus in Uganda, but not a cause of maternal parturitional fevers. We provide cranial imaging that show the progression from meningitis to hydrocephalus in the 1–3 months following initial infection. *Paenibacillus* spp infections are geographically clustered, which is consistent with the hypothesis that this bacteria has an environmental source of infection.

### Implications of all the available evidence

*Paenibacillus* spp is a cause of neonatal sepsis and meningitis as well as postinfectious hydrocephalus in Uganda. Future research that identifies modes of infection, preventive care, and effective treatment is an important next step in reducing the morbidity and mortality associated with *Paenibacillus* spp infections.
