# Supplementary material for: Paenibacillus spp infection among infants with postinfectious hydrocephalus in Uganda: an observational case-control study
Source: Lancet Microbe. Author manuscript; Available in PMC 2023 Sep 27. (PMC10529524; doi:10.1016/S2666-5247(23)00106-4)
Supplement: 1 [file NIHMS1922008-supplement-1.pdf]

THE LANCET
Microbe

Supplementary appendix

This appendix formed part of the original submission and has been peer reviewed. We post it as supplied by the authors.

Supplement to: Morton SU, Hehnly C, Burgoine K, et al. *Paenibacillus* spp infection among infants with postinfectious hydrocephalus in Uganda: an observational case-control study. *Lancet Microbe* 2023; published online June 19. https://doi.org/10.1016/S2666-5247(23)00106-4.

Supplemental Information

Contents | Page

Supplemental Methods	1
Supplemental Table 1: Demographic and Clinical Attributes of Hydrocephalus Group	7
Supplemental Table 2: Participants included in 16S analysis	9
Supplemental Table 3: PIH Participants with and without Paenibacillus by qPCR	10
Supplemental Table 4: Genus Differential Abundance	11
Supplemental Table 5: Species level Differential Abundance	12
Supplemental Table 6: Validation of 16S counts with qPCR as reference	13
Supplemental Table 7: Neonates with Sepsis, Paenibacillus in CSF and Blood	14
Supplemental Table 8: Patients with P. thiaminolyticus CNS infection	16
Supplemental Figure 1: Hydrocephalus Participant Sample Flow Diagram	17
Supplemental Figure 2: Characteristics of 16S dataset	18
Supplemental Figure 3: 16S detection of Paenibacillus·	19
Supplemental Figure 4: Paenibacillus spp and P· thiaminolyticus quantification	20
Supplemental Figure 5: Paenibacillus species annotations with 16S Sequencing	21
Graphical Abstract	22

Supplemental Methods

Study Recruitment

Mother / Newborn Pair Study

Participants in the case-control mother-newborn pair study were recruited at the Mbarara Regional Referral Hospital (RRH) in western Uganda and Mbale RRH in eastern Uganda as previously decribed[2,3]. For both studies, women were ≥18 years of age and able to give informed written consent in a local language. One-hundred (100) women in labor were recruited, 50 from Mbarara RRH and 50 from Mbale RRH. Fifty (50) of the women were febrile at delivery, defined by one oral temperature above 38·1˚C or two of 38·0˚C taken at least an hour apart. Inclusion criteria included delivery at term (>37 weeks estimated gestational age). Women were excluded if there was known intrauterine fetal death, pre-eclampsia, emergency delivery impeding sample collection, antepartum hemorrhage, no telephone number available for post-discharge contact, or domicile >10 km from the hospital.

Neonatal Sepsis Study

For the observational neonatal sepsis study, 800 neonates were recruited, half from Mbale RRH and half from Mbarara RRH as previously decribed[2]. All neonates had an admission weight >2000 grams, and presented with signs of clinical sepsis consistent with a serious bacterial infection. Sepsis was defined as the presence of one of the following three combinations of signs: 1) axillary temperature >37·5 °C, lethargy and poor feeding, 2) axillary temperature <35·5 °C, lethargy and poor feeding, or 3) full fontanelle and/or seizures, axillary temperature >37·5 °C and poor feeding. Inclusion required that women were ≥18 years of age and able to give informed consent in a local language. Neonates were excluded from recruitment if they had major congenital abnormalities, had a history of perinatal asphyxia, or had received antibiotics for more than 24 hours prior to recruitment.

NPIH and PIH Cure Hospital Study

The case-control hydrocephalus study was conducted at the CURE Children's Hospital of Uganda (CCHU) as previously described[1,2], a freestanding pediatric neurosurgical hospital in eastern Uganda that serves as a countrywide referral center for patients with hydrocephalus. Infants were eligible for participation in the study if they < 90 days of age, met the criteria for either postinfectious or non-postinfectious hydrocephalus (PIH and NPIH, respectively), and mothers were ≥18 years of age and able to give informed written consent in a local language. Inclusion criteria for PIH: a) weight >2500 grams, b) no history consistent with hydrocephalus at birth, and either c) a history of febrile illness and/or seizures preceding the onset of clinically apparent hydrocephalus, or d) alternative findings such as imaging and endoscopic results indicative of prior ventriculitis including septations, loculations, or deposits of debris within the ventricular system. Inclusion criteria for NPIH: a) weight > 2500 grams, b) absence of findings consistent with PIH or congenital origin of hydrocephalus and c) findings of non-infectious origin of hydrocephalus on computed tomography (CT) scan or at endoscopy such as a lesion obstructing the Aqueduct of Sylvius such as tumor or cyst, aneurysm, or cavernous malformation, Dandy-Walker cyst, or other congenital malformation of the nervous system, or d) evidence of hemorrhage as cause of hydrocephalus such as bloody CSF and absence of findings consistent with PIH or congenital origin of hydrocephalus. Exclusion Criteria: a) prior surgery on the nervous system (shunt, third ventriculostomy, or myelomeningocele closure), or b) evidence of communication of nervous system with skin such as meningocele, encephalocele, dermal sinus tract, fistula, etc. Cases and controls were not explicitly matched on any demographic or clinical criteria other than the inclusion/exclusion criteria.

Sample collection

Samples were divided for fresh freezing or placement into DNA/RNA Shield (Zymo Corporation), and all specimens were then stored at -80°C. Participants recruited into the maternal-newborn pair cohort had four sample types that were collected in DNA/RNA Shield or fresh frozen: intrapartum maternal blood and vaginal swab, postpartum cord blood and fetal-side placenta. Participants recruited into the neonatal sepsis study had both blood and CSF samples that were preserved in DNA/RNA Shield and fresh frozen.

DNA Extraction, qPCR and 16S Sequencing

Nucleic acid extraction was performed as previously described[1]. Briefly, 500 µL of the sample stored in DNA/RNA Shield was extracted using ZymoBIOMICS DNA Miniprep Kit (Zymo, CA, USA) following the manufacturer's instructions with bead lysis, proteinase K treatment and eluted in 100 µL of heated elution buffer. The 16S rDNA V1-V2 region was amplified and sequenced[4]. Briefly, primer-extension polymerase chain reaction (PE-PCR) of the 16S rRNA hypervariable regions was performed[4] with region-specific primers. Specifically, 2 µl of DNA was mixed with 2 ng/µl the V1-V2 region annealing probe 336R-M13 (5'-CAGGGTTTTCCCAGTCACGACACTGC-TGCSYCCCGTAGGAGTCT-3'), 10X buffer and water then extended with 5 U/µl Klenow (NEB, Cat# M0210) and excess primers digested with 20 U/µl exonuclease (NEB, Cat# M0293). The extended product was amplified with 500 nM of 27F (AGAGTTTGATCMTGGCTCAG) and M13 (5'-CAGGGTTTTCCCAGTCACGAC -3') with MolTaq 16S/18S PCR 2X mastermix (Molzym, Cat# S-030-0250) for 35 cycles of 95 °C for 15 seconds and 60 °C for two minutes. For library preparation, amplified product was cleaned and the Hyper Prep Kit library preparation kit (KAPA Biosystems, Cat# KK8504) was used following the manufacturer's protocols with seven cycles of library amplification. Libraries were quantified with Qubit or Agilent Bioanalyzer DNA 1000 Chip and sequenced on Illumina's MiSeq using the 600 cycle v3 kit with 20% PhiX and 6pM pooled library.

For qPCR, 10 µl reactions of 2 µl of DNA mixed with 2X TaqMan gene expression mastermix (Applied Biosystems, Cat# 4369016) and 200 nM of primers and probes for the 16S *Paenibacillus* genus previously described[5] or in house designed thiaminase gene primers for *P. thiaminolyticus* (Fwd: 5'-GGGCGAGTATGCGGATCA-3' ; Rev: 5'-CTGCCCCGCCGATGA-3'; Probe: 5'-FAM-CAGTTCAAGACGATTTC-MGB-3') were amplified for 45 cycles of 95°C for 15 seconds and 60 °C for 90 seconds after an initial 10 minute enzyme activation on a QuantStudio Real-Time 12K Flex PCR instrument (Thermo, USA). A standard curve of genomic DNA or PCR template vector diluted from 500,000 to five copies/µl was used for quantification. Samples were considered positive if two of three technical replicates were positive. For qPCR of cytomegalovirus, it was performed as previously described[2].

For filtering of the 16S sequencing, ASVs mapping to the family mitochondria or order chloroplast were removed, as were contaminants and ASVs that were unmapped at the phylum level. Furthermore, 2179 ASVs that were mapped to Proteobacteria without further classification were clustered into 55 clusters, and the dominant sequence from each cluster was mapped onto human protein isoforms. These 2179 ASVs within clusters that mapped to human proteins were removed. The Bioconductor package MicrobiomeExplorer was used for subsequent analysis[6]. ASVs present in fewer than 10 samples, or observed at a depth of less than 100, were filtered out. Samples with fewer than 10 bacterial organisms or fewer than 100 reads were also filtered out. Data were then normalized by aggregation at the genus level and calculation of per-genus read proportions for each sample. After filtering, 201 PIH and 178 NPIH samples were included in the analysis. This remained true after leveraging novel approaches to account for spurious technical artifacts and when samples with *Paenibacillus* detected by qPCR were removed[7]. Pairwise distance of the ASV sequences was performed with DECIPHER[8] using the AlignSeqs function with default settings

Removal of Contaminating Sequences

16S sequencing data were processed to identify likely environmental contaminants using the decontam R package (https://benjjneb.github.io/decontam/vignettes/decontam_intro.html). No Paenibacillus taxa were flagged by decontam as contaminants in the overall dataset. To reduce the high loads of spurious taxa[7,9] in microbiome data, before downstream analysis, taxa correlated in their abundance across all samples were clustered together using distribution-based clustering[10].

Statistical analysis

For categorical variables, chi-squared or Fisher exact tests were used to compare groups. Correlations between two continuous variables were assessed using Pearson's correlation. Values are expressed as the mean ± standard deviation (SD), median and interquartile range in case of a skewed distribution, and as counts and percentages for categorical variables. Normality assumption for continuous variables was tested using the Shapiro-Wilk test[11]. Confidence intervals (95% CI) for prevalence were estimated using an exact binomial test. Univariate and multivariate binary logistic regression was used to evaluate risk factors. We report the odds ratios (OR) and 95% CI for the odds of *Paenibacillus* infection. Statistics for 16S and qPCR comparisons were done using caret confusionMatrix() (https://topepo.github.io/caret/).

Brain Computed Tomography Scans

Preoperative computed tomography (CT) brain scans were scored as previously described[1]. Briefly, the scans were independently scored blindly with respect to diagnosis by two physicians with considerable experience interpreting hydrocephalic infant CT scans (P.S. and J.L). A point

was assigned for each of the four possible findings: 1) fluid loculations, 2) debris within fluid spaces, 3) ectopic calcifications within the brain parenchyma, or 4) abscess formation. Scoring discrepancies were resolved by consensus agreement.

References

1. Paulson JN, Williams BL, Hehnly C, et al. Paenibacillus infection with frequent viral coinfection contributes to postinfectious hydrocephalus in Ugandan infants. *Sci Transl Med* 2020; **12**(563).
2. Movassagh M, Bebell LM, Burgoine K, et al. Vaginal microbiome topic modeling of laboring Ugandan women with and without fever. *NPJ Biofilms Microbiomes* 2021; **7**(1): 75.
3. Hehnly C, Ssentongo P, Bebell L, et al. Cytomegalovirus Infections in Ugandan Infants: Newborns, Neonates with Sepsis and Infants with Hydrocephalus.
4. Chang SS, Hsu HL, Cheng JC, Tseng CP. An efficient strategy for broad-range detection of low abundance bacteria without DNA decontamination of PCR reagents. *PLoS One* 2011; **6**(5): e20303.
5. Bolyen E, Rideout JR, Dillon MR, et al. Reproducible, interactive, scalable and extensible microbiome data science using QIIME 2. *Nature Biotechnology* 2019; **37**(8): 852-7.
6. Salter SJ, Cox MJ, Turek EM et al. Reagent and laboratory contaminants can critically impact sequence-based microbiome analyses. *BMC Biology* 2014; **12**: 87.
7. Kumar MS, Slud EV, Hehnly C, et al. Differential richness inference for 16S marker gene surveys. *Genome Biology* 2022; **23**: 166.
8. Preheim SP, Perrotta AR, Martin-Platero AM, et al. Distribution based clustering: using ecology to refine the operational taxonomic units. *Appl Environ Microbiol* 2013; **79**(21): 6593-6603.

Supplemental Table 1: Demographic and Clinical Attributes of Hydrocephalus Group

	All Patients (n=400)	PIH (n=209)	NPIH (n=191)
Age at presentation of initial infection, days, mean (SD)	NA	8·0 (7·0)	NA
Age at sample collection, days, mean (SD)	50·7 (24·9)	61·8 (17·4)	38·5 (26·3)
Days between infection and collection, mean (SD)	NA	51·5 (18·5)	NA
Sex<br>Female N(%)<br>Male N(%)	 173 (44)<br>227 (56)	 91 (44)<br>118 (56)	 82 (43)<br>109 (57)
Blood WBC, cells x10^3 per microliter, mean (SD)	10·3 (3·3)	10·9 (3·2)	9·6 (3·3)
CSF WBC, cells per microliter, mean (SD)	25·2 (57·7)	42·5 (74·0)	6·3 (17·8)
Hgb, g/dL, mean (SD)	12·0 (2·8)	10·6 (1·6)	13·6 (2·9)
HIV exposure, % (n)	2·3 (9)	2·4 (5)	2·1 (4)
Blood CMV positive, % (n)	22·3 (89)	24·9 (52)	19·4 (37)*
CSF CMV positive, % (n)	7·0 (27)	12·4 (26)	0·5 (1)*
CT score, # (%)			
0	150 (38)	27 (13)	123 (64)
1	95 (24)	48 (23)	47 (25)
2	49 (12)	40 (19)	9 (5)
3	40 (10)	40 (19)	0 (0)
4	50 (13)	49 (23)	1 (<1)
Missing	16 (4)	4 (2)	12 (6)

Abbreviations and Definitions: CMV, cytomegalovirus; CSF, cerebrospinal fluid; CT, computed tomography; Hgb, hemoglobin; HIV, human immunodeficiency virus; NPIH, non-postinfectious hydrocephalus; PIH, postinfectious hydrocephalus; SD, standard deviation; WBC, white blood cell count. * One NPIH participant had unknown blood/CSF CMV status.

Supplemental Table 2: Participants included in 16S analysis			
Characteristic	PIH (n=201)	NPIH (n=178)	p-value
Age at initial infection, days, mean (SD)	7·9 (7·1)	NA	NA
Age at sample collection, days, mean (SD)	62·1 (17·2)	38·9 (26·5)	**<2·2E-16**
Days between infection and collection, mean (SD)	52·1 (18·3)	NA	NA
Sex, % female (n)	43·3 (87)	42·1 (75)	1
Blood WBC, x10^3, mean (SD)	10·8 (3·2)	9·7 (3·3)	**1·71E-04**
CSF WBC, mean (SD)	39·3 (71·2)	6·5 (18·4)	**2·04E-07**
Hgb, mean (SD)	10·6 (1·6)	13·5 (2·8)	**<2·2E-16**
HIV exposure, % (n)	2·0 (4)	1·7 (3)	1
Blood CMV positive, % (n)	24·9 (50)	20·2 (36)	1
CSF CMV positive, % (n)	11·9 (24)	0·6 (1)	**8·67E-06**
CT score, #			
0	27	113	**1·70E-16**
1	47	44	
2	38	9	
3	38	0	
4	46	1	
Missing	5	11	

Supplemental Table 3: PIH Participants with and without Paenibacillus detected by genus qPCR			
Characteristic	PIH with Paenibacillus (n=98)	PIH without Paenibacillus (n=110)	p-value
Age at initial infection, days, mean (SD)	7·2 (6·0)	8·7 (7·8)	2·50E-01
Age at sample collection, days, mean (SD)	57·5 (16·6)	65·5 (17·1)	**5·02E-04**
Days between infection and collection, mean (SD)	50·8 (16·3)	58·2 (17·2)	**3·86E-03**
Sex, %female (n)	45·9 (45)	40·9 (45)	1
Blood WBC, x10^3, mean (SD)	11·6 (3·6)	10·4 (2·8)	**1·84E-02**
CSF WBC, mean (SD)	66·7 (85·5)	20·5 (53·9)	**1·92E-08**
Hgb, mean (SD)	10·6 (1·7)	10·4 (1·5)	8·15E-01
HIV exposure, % (n)*	1·1 (1)	3·9 (4)	1
Blood CMV positive, % (n)	21·4 (21)	28·1 (31)	1
CSF CMV positive, % (n)	13·3 (13)	11·8 (13)	1
CT score, #			
0	5	22	2·49E-01
1	15	33	
2	20	19	
3	22	18	
4	33	16	
Missing	1	2	

*HIV exposure status missing from 2 *Paenibacillus* positive and 7 negative participants

.

Supplemental Table 4: Genus Differential Abundance			
Genus	Average NPIH Counts	Log2 Counts (PIH/NPIH)	p-value
Paenibacillus	1·957	3·359	1·18E-18
Cutibacterium	7·399	-1·668	2·68E-12
Rickettsiales	2·298	1·761	1·44E-08
Anaerococcus	0·292	-0·53	1·23E-05
Corynebacterium	2·762	-1·388	4·05E-05
Alphaproteobacteria	0·744	0·804	9·85E-05
Marinomonas	0·418	-0·697	7·95E-05
Brochothrix	0·442	-0·664	1·31E-04
Psychromonas	0·319	-0·523	1·31E-04
Sphingomonas	1·617	-1·023	1·75E-04
OPB56	0·172	-0·33	2·12E-04
Peptoniphilus	0·305	-0·385	1·23E-03
Meiothermus	0·2	-0·278	1·55E-03
Tepidimonas	1·161	0·863	4·27E-03
Staphylococcus	4·29	-1·038	2·42E-03
Lawsonella	0·831	-0·563	5·37E-03
Kocuria	0·92	-0·57	9·47E-03
Massilia	0·985	-0·704	9·47E-03
Hydrogenophilus	0·566	-0·436	9·47E-03

Supplemental Table 5: Species level Differential Abundance			
Species	Average NPIH Counts	Log2 Counts(PIH/NPIH)	p-value
Paenibacillus popilliae	1·72	3·081	2·73E-18
Paenibacillus thiaminolyticus	1·387	2·384	5·04E-14
Corynebacterium kroppenstedtii	0·955	-1·5	2·32E-10
Cutibacterium granulosum	0·718	-0·856	9·72E-06
Sphingomonas leidyi	1·499	-1·048	1·12E-04
Hydrogenophilus islandicus	0·459	-0·561	1·26E-04
Pseudomonas luteola	0·187	-0·353	1·26E-04
Meiothermus silvanus	0·2	-0·278	2·11E-03
Staphylococcus capitis	1·405	-0·817	3·33E-03
Corynebacterium macginleyi	0·109	-0·216	6·35E-03
Kocuria rhizophila	0·137	-0·22	8·52E-03
Corynebacterium suicordis	0·317	-0·317	1·77E-02
Massilia aurea	0·747	-0·556	2·59E-02
Corynebacterium tuberculostearicum	1·708	-0·643	2·98E-02
Cutibacterium acnes	0·175	-0·137	3·04E-02
Hymenobacter rigui	0·098	-0·189	3·72E-02
Staphylococcus epidermidis	2·837	-0·723	4·43E-02
Pseudomonas psychrotolerans	0·11	-0·19	4·43E-02

Supplemental Table 6: Validation of 16S counts with qPCR as reference

	Paenibacillus spp.	***P. thiaminolyticus***
Sensitivity, % (95% CI)	84 (75-91)	77 (67-86)
Specificity, % (95% CI)	97 (94-99)	98 (95-99)
Positive Predictive Value , % (95% CI)	90 (81-95)	90 (81-96)
Negative Predictive Value , % (95% CI)	95 (92-97)	94 (91-96)
Overall Accuracy, % (95% CI)	94 (91-96)	93 (90-95)

Abbreviation: CI, confidence interval

Supplemental Table 7: Neonates with Sepsis *Paenibacillus* status in Cerebrospinal Fluid and Blood					
Study ID	Cerebrospinal Fluid		Blood		Hydrocephalus
qPCR target	Genus	Species	Genus	Species	
1192	Negative	Negative	Negative	Positive	
1245	Negative	Negative	Negative	Positive	
1265	Positive	Positive	Negative	Negative	
1276	Positive	Negative	Negative	Negative	
1279	Positive	Positive	Negative	Negative	
1281	Positive	Positive	Negative	Negative	
1288	Positive	Negative	Negative	Negative	
1296	Positive	Negative	Negative	Negative	
1301	Positive	Positive	Negative	Negative	
1311	Positive	Positive	Negative	Negative	
1316	Positive	Positive	Negative	Negative	
1317	-	-	Negative	Positive	
1321	Positive	Negative	Negative	Negative	
1323	Positive	Negative	Negative	Negative	
1329	Positive	Negative	Negative	Negative	
1348	Positive	Positive	Negative	Negative	
1351	Positive	Positive	Negative	Negative	
1362	Positive	Positive	Negative	Negative	
1371	Positive	Positive	Negative	Negative	
1376	Positive	Positive	-	-	
1394	Positive	Positive	Negative	Negative	
1396	Positive	Positive	Negative	Negative	
3108	Positive	Positive	Negative	Negative	Referred, didn't show
3121	Positive	Negative	Negative	Negative	
3128	Positive	Negative	Negative	Negative	
3168	Positive	Negative	Negative	Negative	
3176	Negative	Positive	Negative	Negative	Y, compensated
3183	Negative	Positive	Negative	Negative	
3323	Positive	Positive	Negative	Negative	Y, recruited, PIH
3334	Positive	Positive	Negative	Negative	Y, recruited, PIH
3369	Positive	Positive	Negative	Negative	Y, compensated
3376	Positive	Positive	Negative	Negative	
3377	Positive	Positive	Positive	Positive	
3382	Positive	Positive	Negative	Negative	
3385	Positive	Negative	Negative	Negative	

3391	Positive	Negative	Negative	Negative	
3394	Positive	Positive	Negative	Negative	
3397	Positive	Positive	Negative	Negative	

Abbreviations: -, no sample; Y, yes; PIH, postinfectious hydrocephalus

Supplemental Table 8: Summary of the clinical and demographic data for patients with *P. thiaminolyticus* CNS infection that developed PIH

	Patient 1		Patient 2	
**Infant factors**	*NS*	*PIH*	*NS*	*PIH*
Sex	Male		Male	
Treatment site	Mbale RRH	CURE	Mbale RRH	CURE
Age in days	8	44	7	73
Temperature,□C	38·3	-	37·0	
Weight, kg	3·06	-	2·00	
Respiratory rate, breath per minute	51	-	67*	
Oxygen saturations %	96	-	66^	
Heart rate, beats per minute	148	-	138	
Blood, WBC/µL	-	14400	-	8800
Hematocrit, %	-	45·1	-	36·5
Hemoglobin, g/dL	-	12·8	-	11·0
Granulocytes, %	-	42·2	-	31·0
Lymphocytes, %	-	50·1	-	58·7
CSF Glucose, mmol/L	0	20	0	36
CSF Proteins, mg/dL	180*	300*	50	100*
CSF, WBC/µL	75*	200*	80*	45*
Head circumference, cm (AAP)	35·7 (63·8)	37·2 (34·2)	33·5 (13·6)	42·2 (97·2)
Seizures	Present	-	Present	-
Jaundice	No	-	Yes	-
**Maternal factors**				
Age in years	29		23	
Febrile during labor	Yes		Yes	
HIV Status	Negative		Negative	
Place of Delivery	Home		Healthcare Facility	

Abbreviation: NS, Neonatal Sepsis; PIH, postinfectious hydrocephalus; Mbale, Mbale Regional Hospital; AAP, age adjusted percentile based on https://www.infantchart.com/infantheadage.php
*abnormal high; ^ abnormal low

Participant Sample Flow Diagram

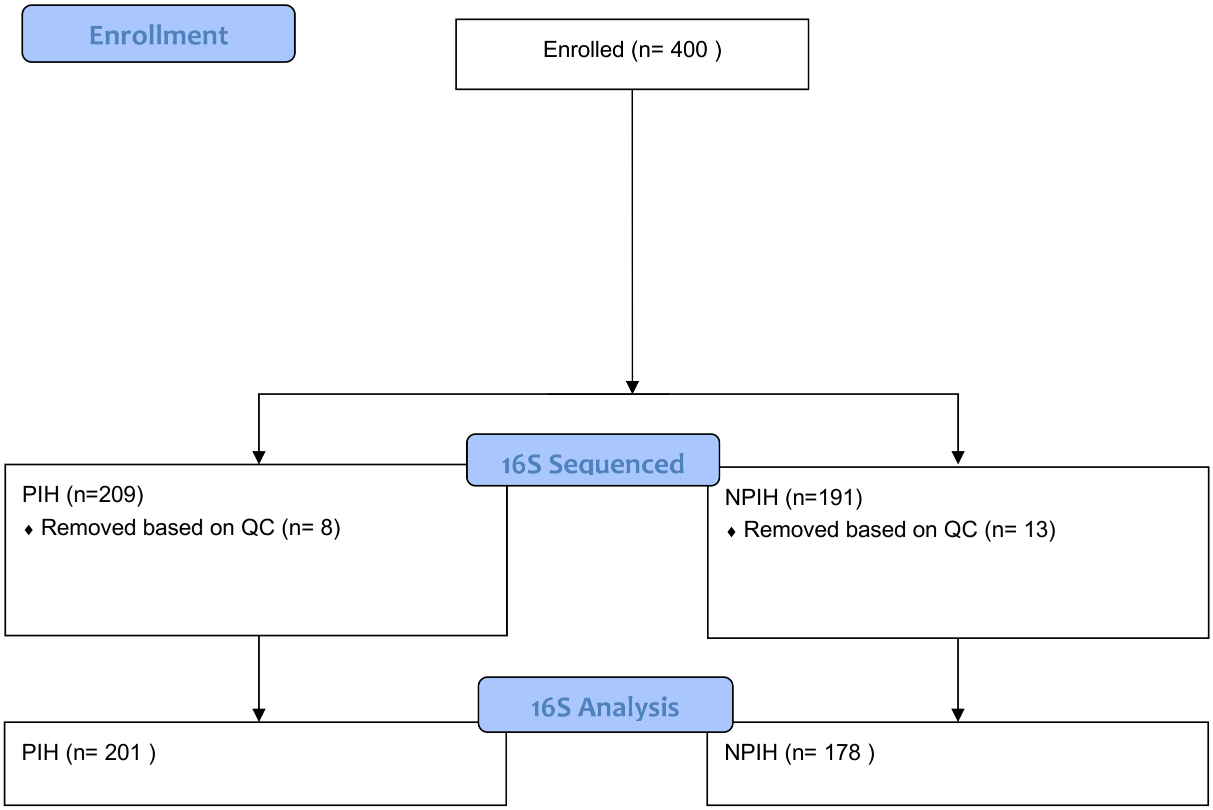

Supplemental Figure 1: Hydrocephalus Participant Sample Flow Diagram

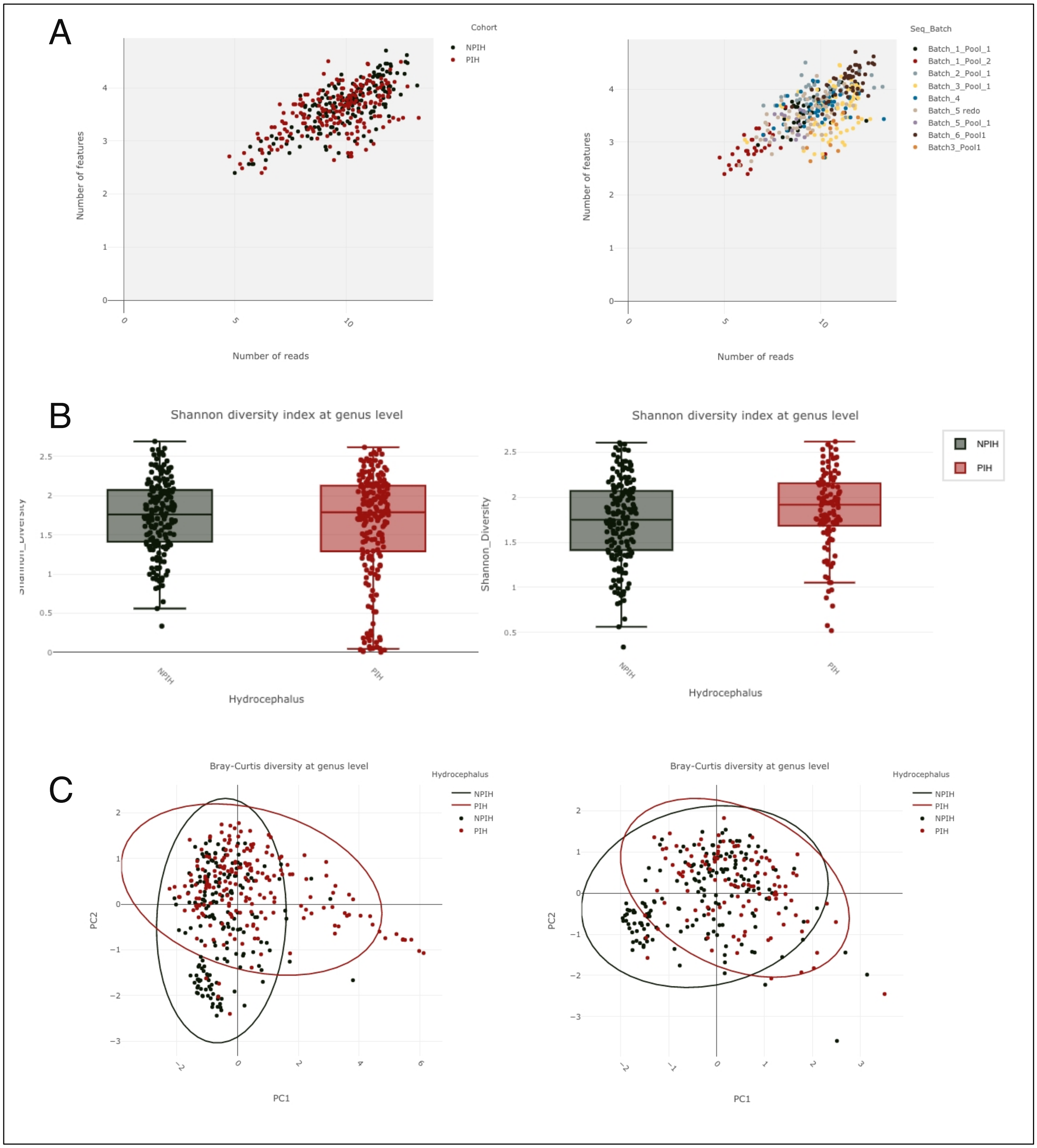

***Supplemental Figure 2: Characteristics of 16S dataset*·** The quantification of mapped 16S reads per participant A) relative to number of features when subset by cohort (left) or sequencing batch (right); B) compared using Genus-level Shannon diversity index for the whole cohort (left) or with samples positive for *Paenibacillus* by qPCR removed (right); C) or Genus-level Bay-Curtiss diversity index for the whole cohort (left) or with samples positive for *Paenibacillus* by qPCR removed (right)·

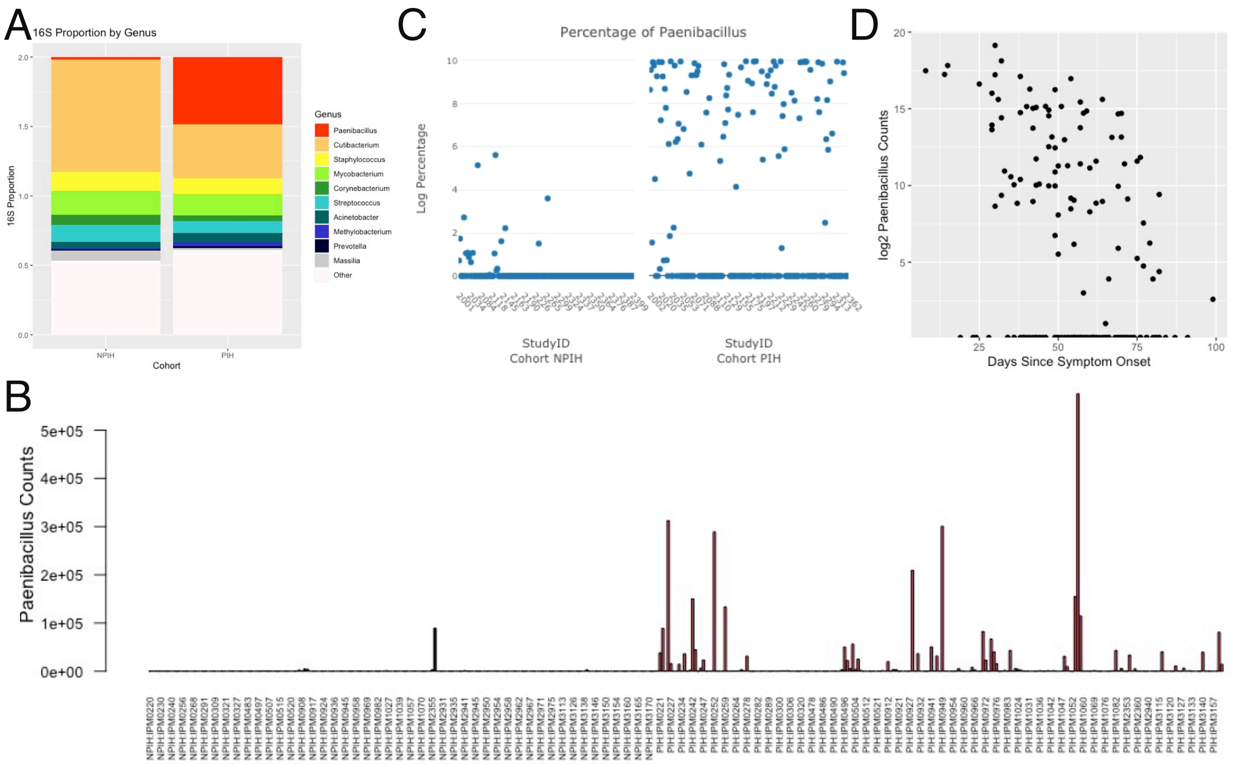

***Supplemental Figure 3: 16S detection of Paenibacillus*·** A) Comparison of top 10 most abundant genera in the NPIH and PIH cohorts· B) *Paenibacillus* 16S counts per sample· C) Log percentage of *Paenibacillus* as a proportion of total reads per sample· D) Log2 *Paenibacillus* 16S counts per sample relative to days between sample collection and symptom onset·

A B

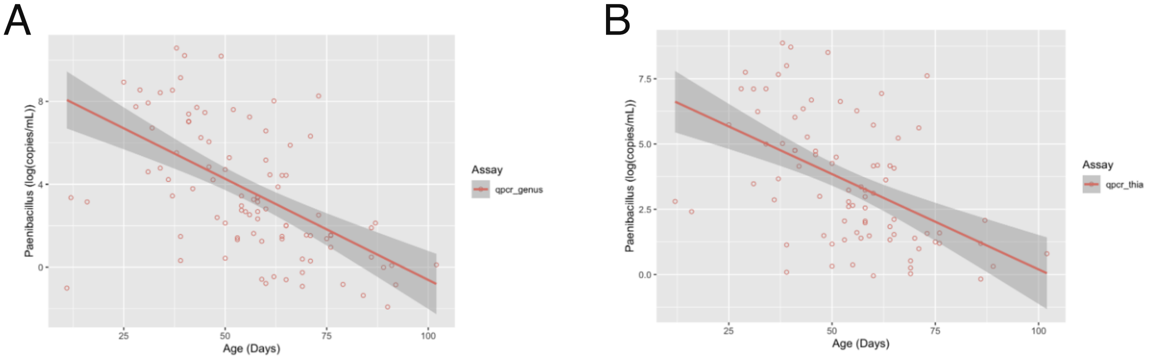

C

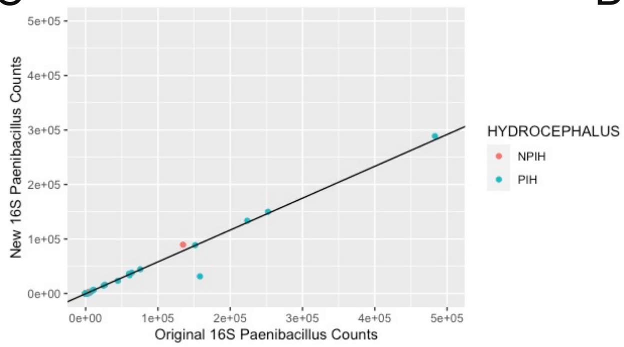

D

PIH, n=62	Paenibacillus >307.5 Counts	Paenibacillus <307.5 Counts
Paeni >50 First Cohort	32	5
Paeni <50 First Cohort	0	25

NPIH, n=35	Paenibacillus >307.5 Counts	Paenibacillus <307.5 Counts
Paeni >50 First Cohort	2	0
Paeni <50 First Cohort	0	33

Supplemental Figure 4: Paenibacillus spp and P· thiaminolyticus quantification by age and comparison to original publication· The quantification of *Paenibacillus* by qPCR of A) *Paenibacillus* spp· B) *P· thiaminolyticus* by qPCR as a function of age· C) Comparison of 16S *Paenibacillus* counts from original cohort and current analysis. D) Comparison of *Paenibacillus* status as determined by 16S reads from original cohort and current analysis.

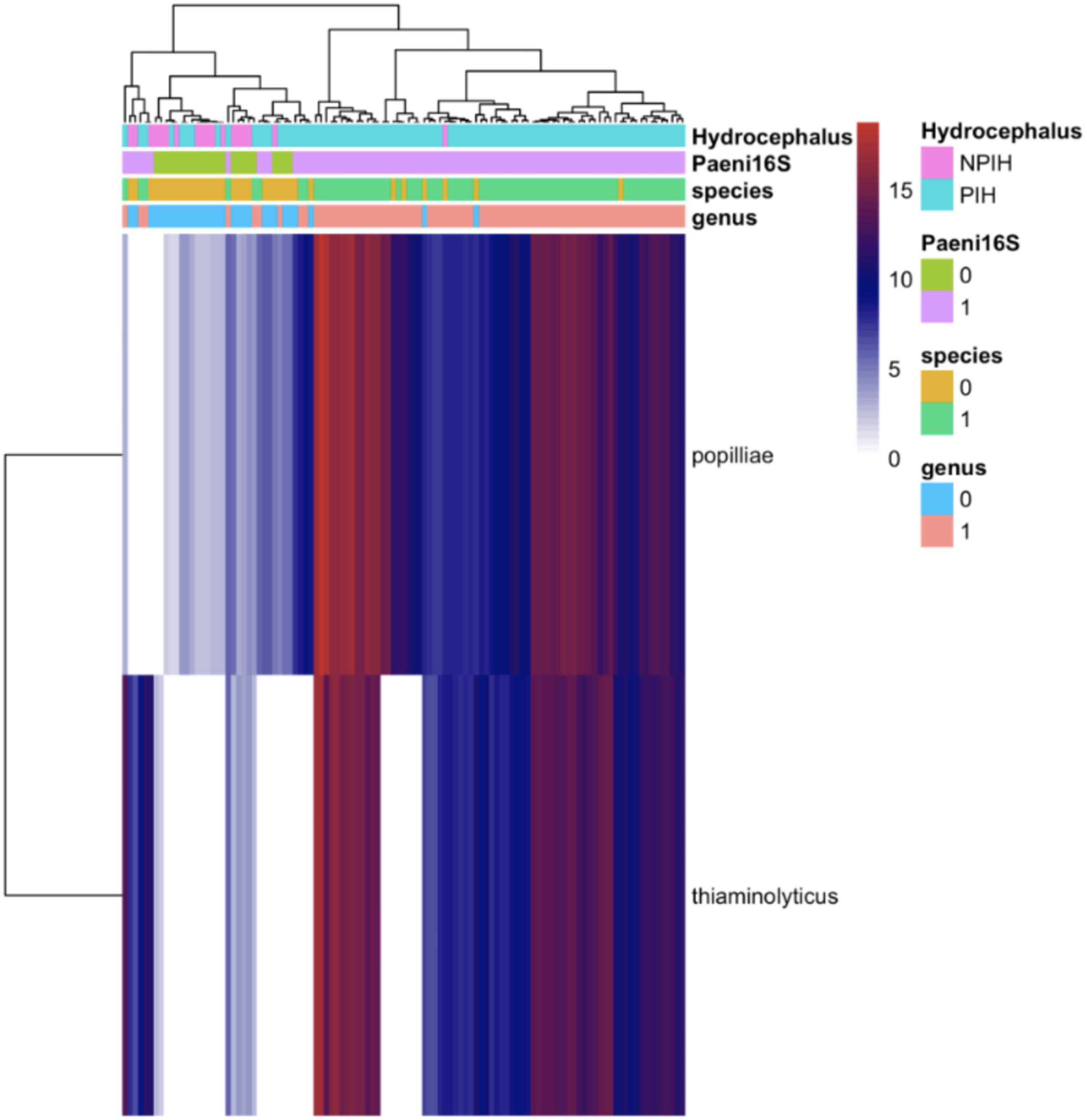

Supplemental Figure 5: Paenibacillus species annotations with 16S Sequencing.
A heatmap of the summarized amplicon sequence variant (ASV) counts of each species of *Paenibacillus* annotated with qPCR genus or species-level detection status, 16S status and hydrocephalus status

WHAT BACTERIA ARE ASSOCIATED WITH INFANT POSTINFECTIOUS HYDROCEPHALUS IN UGANDA?

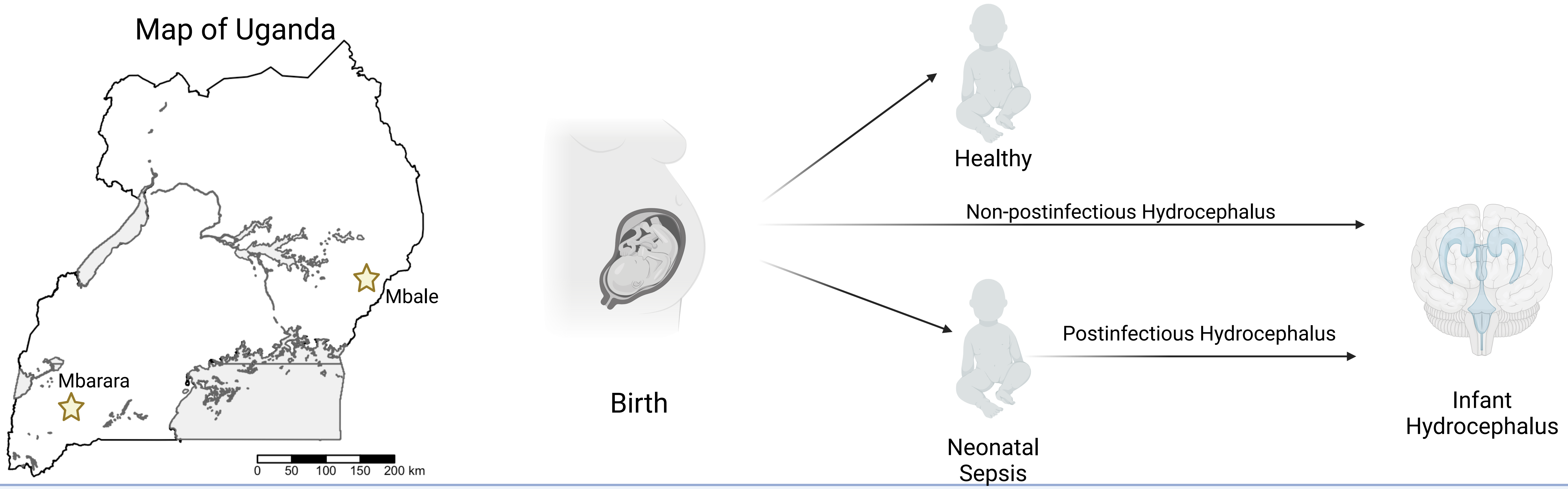

REPRESENTATIVE COHORTS

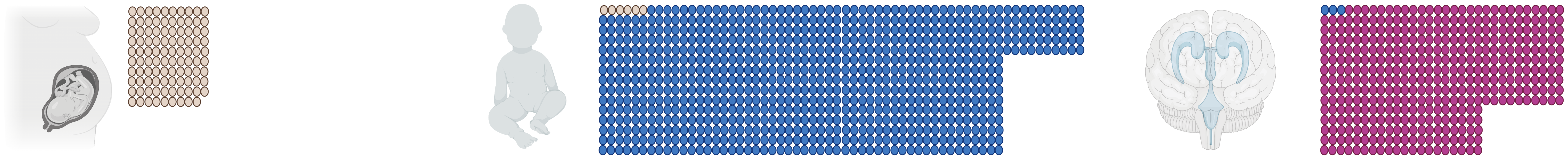

Maternal-Newborn Pairs (n=99) Neonatal Sepsis (n=800) **Infant Hydrocephalus (n-400)**

METHODS

Unbiased 16S rDNA Metagenome Sequencing

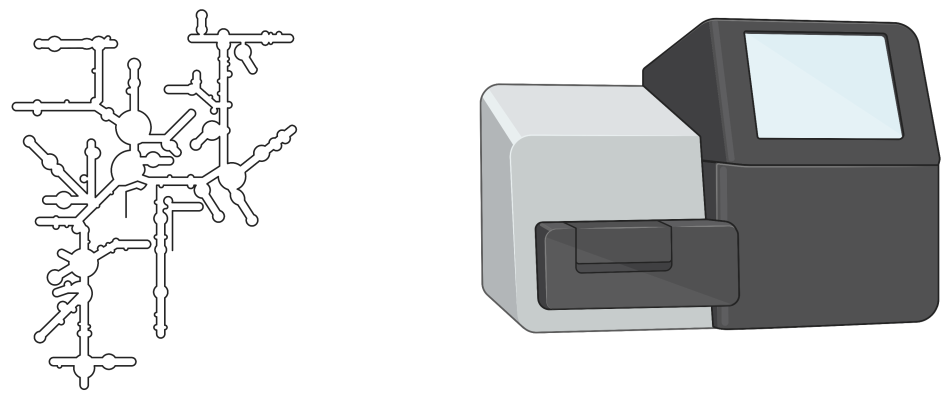

Infant Hydrocephalus

Targeted quantitative PCR to *Paenibacillus*

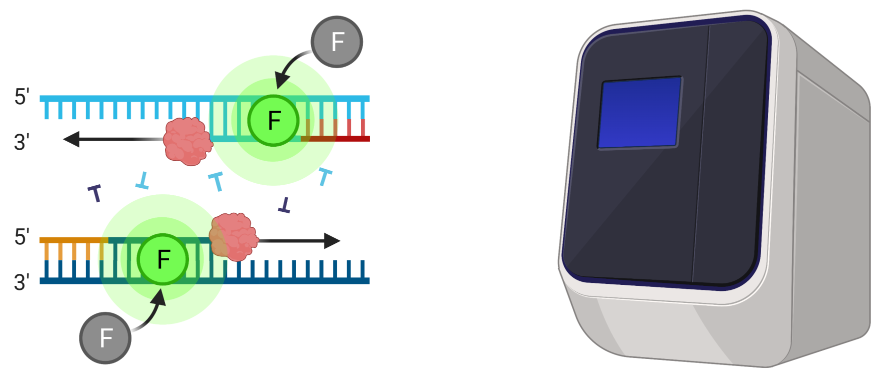

Maternal-Newborn Pairs - Neonatal Sepsis - Infant Hydrocephalus
